# Age‐Related Differences in Neural Correlates of Auditory Spatial Change Detection in Real and Virtual Environments

**DOI:** 10.1111/ejn.70141

**Published:** 2025-05-15

**Authors:** Benjamin Stodt, Daniel Neudek, Rainer Martin, Edmund Wascher, Stephan Getzmann

**Affiliations:** ^1^ Leibniz Research Centre for Working Environment and Human Factors at the TU Dortmund (IfADo) Dortmund Germany; ^2^ Institute of Communication Acoustics Ruhr‐Universität Bochum Bochum Germany

**Keywords:** aging, mismatch negativity, P3b, spatial hearing, virtual reality

## Abstract

Although virtual environments are increasingly used in research, their ecological validity in simulating real‐life scenarios, for example, to investigate cognitive changes in aging populations, remains relatively unexplored. This study aims to evaluate the validity of a virtual environment for investigating auditory spatial change detection in younger and older adults. This evaluation was performed by comparing behavioral and neurophysiological responses between real and virtual environments. Participants completed an auditory change detection task, identifying sound source position changes relative to a reference position. In the real environment, sounds were presented through physical loudspeakers in a reverberant room. In the virtual environment, stimuli were delivered through headphones, accompanied by a head‐mounted display showing a visual replica of the room. Participants showed higher accuracy for azimuth than for distance changes, regardless of age or environment, emphasizing humans' larger sensitivity to lateralized sounds. Event‐related potentials were mostly consistent across environments, with significantly higher N1 and P2 amplitudes in older compared with younger adults. Mismatch negativity was reduced in older adults, and both reduced and delayed in the virtual environment. The P3b showed larger amplitudes and shorter latencies for azimuth changes, reflecting greater salience of directional cues, whereas responses in the virtual environment were slightly diminished, especially among older adults. Bayesian analyses validated the observed effects. Results support virtual environments as reliable tools for exploring spatial perception and underlying neural and behavioral processes in realistic contexts. Furthermore, differences in the processing of spatial changes in azimuth and distance, as well as age‐related effects, could be highlighted.

## Graphical abstract

We examined auditory change detection in real and virtual environments across two age groups using behavioral measures and event‐related potentials. Participants responded to changes in distance and azimuth from a central reference loudspeaker. Results underscore the potential of audiovisual virtual environments as a research tool for exploring auditory processing and associated neural mechanisms.

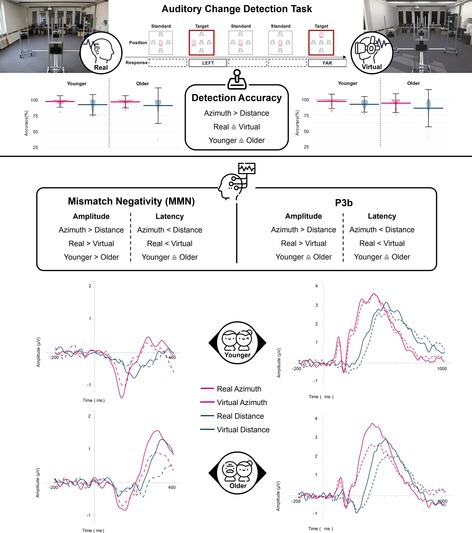

**Abbreviations**
Ag/AgCl: silver/silver chloride
ANOVA: analysis of variance
BF: Bayesian factor
BRIR: binaural room impulse response
*d*_*c*_
: critical distance
EEG: electroencephalogram
ERP: event‐related potential
FAL: fractional area latency
*f*_*S*_
: sampling frequency
H_0_
: null hypothesis
H_1_
: alternative hypothesis
*h*_*LS*_
: height of loudspeaker
*h*_*p*_
: height of participants' ears
HMD: head‐mounted display
IC: independent component
ICA: independent component analysis
MMN: mismatch negativity
*T*_60_
: reverberation time
VR: virtual reality

## 1 Introduction

Virtual reality (VR) has evolved into a powerful tool, playing a central role not only for entertainment purposes but also in psychological research (de la Rosa and Breidt 2018; Vasser and Aru 2020). Here, its applications are already varied, encompassing areas such as mental health, social interaction, and training, with expectations for an even broader usage in the future (Martingano and Persky 2021; Rizzo and Koenig 2017; Vasser and Aru 2020). One advantage is that researchers can create authentic representations of scenarios or environments that are difficult to replicate in real‐world settings, while still being able to control all aspects of the experimental environment and ensuring consistent conditions across trials. It also enables easy modification and adaptation of the environment and experimental tasks to test different hypotheses. Furthermore, the use of VR can reduce costs, as experiments can be conducted more efficiently and without the need for expensive physical equipment (de la Rosa and Breidt 2018; Rizzo and Koenig 2017). It has been shown that VR enhances attention abilities compared with traditional laboratory setups, such as those using computer screens for stimulus presentation (Li et al. 2020). Therefore, VR offers a promising solution to the challenge of ecological validity in conventional laboratory studies, which often struggle to accurately represent real‐world conditions and applications (Parsons 2015). Although the experimental tasks examined in VR are often simplified compared with real‐world tasks, they provide greater ecological validity and help bridge the gap between realism and experimental control (Parsons 2015).

Recent studies have already investigated mechanisms of spatial auditory perception and attention using virtual acoustic reproductions of real environments (Blau et al. 2021; Bosman et al. 2024; Enge et al. 2020; Neidhardt et al. 2022; Van De Par et al. 2022). Although the neural processes involved in auditory localization and attention to sound sources varying in azimuth are well‐studied (e.g., Altman et al. 2005; Deouell et al. 2006; Getzmann and Näätänen 2015; Lewald and Getzmann 2011), perception of sound sources varying in their distance to the listener remains relatively unexplored (Andreeva et al. 2024; Kolarik et al. 2016; Kopčo et al. 2012; Mathiak et al. 2003; Paquier et al. 2016). Humans use binaural cues, such as interaural time and level differences, which are particularly effective for the perception and localization of sound sources distributed in azimuth. Studies show that although sound sources positioned in azimuth can be localized with high accuracy using binaural cues, distance perception is less accurate, especially for nearby sources where these cues are less distinct (Arend et al. 2021). Because of the unreliability of these cues, individuals frequently underestimate proximity (Andreeva et al. 2024). To generate an integrated perception of auditory distance, other acoustic properties of the sound reaching the human ear become relevant. Sound intensity is the most important one as intensity decreases as the distance to the sound source increases (Kolarik et al. 2016; Zahorik et al. 2005). However, this is only the case if the source intensity remains constant and the listener is familiar with both the sound source and the environment. For unfamiliar sounds or varying intensities, additional cues like the direct‐to‐reverberant energy ratio (DRR), which decreases with distance, and changes in spectral characteristics also play a role (Kopčo and Shinn‐Cunningham 2011; Spiousas et al. 2017; Zahorik et al. 2005).

In a virtual acoustic environment, a full integration of binaural and distance‐dependent auditory cues facilitates a realistic perception and interaction with the environment, which in turn can enhance the ecological validity of research findings (Bosman et al. 2024). However, several factors may also limit the accuracy of spatial perception in virtual acoustic environments. First, virtual binaural audio relies on head‐related transfer functions (HRTFs) to simulate auditory cues, but unless individualized HRTFs are used, mismatches between the listener's actual and simulated auditory filtering can lead to localization inaccuracies (Jenny and Reuter 2020). Additionally, uncertainties in the modeling (e.g., recording of binaural room impulse responses or headphone transfer functions) could lead to losses of spatial detail, reducing the reliability of the simulated environment (Zahorik 2009). Further, although real environments allow for dynamic listener interactions, many virtual scenarios remain largely static. Such limited physical engagement may reduce both the realism of spatial cues and the sense of immersion, particularly when head movements are not implicitly integrated into the auditory rendering (Brimijoin et al. 2013). These factors highlight the importance of further investigating the extent to which virtual auditory environments can accurately replicate real‐world spatial scenarios.

Furthermore, in auditory‐only investigations, significant limitations often arise from the lack of sufficient visual information, which is essential for everyday listening scenarios (Van De Par et al. 2022). Incorporating visual references into auditory tasks (such as cues related to room size and depth) can profoundly influence cognitive processing and response efficiency. This underscores the necessity of integrating multiple sensory modalities in both research and practical applications. Multimodal approaches are particularly valuable for exploring how auditory and visual systems interact in contexts that demand heightened attention, such as conversations and work tasks (Breuer et al. 2022; Hohmann et al. 2020). When visual objects are accurately represented, they enhance the overall impression and intensify the sense of presence by directing the listener's attention (Best et al. 2007).

As an intermediate conclusion, it is essential to develop virtual environments that accurately replicate the most salient environmental characteristics necessary for performing specific tasks in realistic scenarios. Furthermore, it is important to ensure that the results collected in these virtual environments are plausible and consistent with those obtained in real‐world settings or measured with the same level of accuracy as in laboratory conditions. Validation could involve comparing the outcomes of standardized experimental tasks performed in both real and virtual test environments, where consistent results across both settings would indicate a high validity of the virtual environment (Stodt et al. 2024). Additionally, this approach could provide insights into which specific characteristics are essential for optimal performance in the virtual environment and whether there are interindividual differences in the two settings.

An important individual factor that influences auditory processing is aging, potentially impacting daily life activities due to its links with hearing impairment and cognitive decline (Tun et al. 2012). Research indicates that older adults face challenges in auditory processing compared with younger adults. As aging can affect both peripheral and central auditory functions, this could lead to difficulties in sound localization and temporal processing (Dobreva et al. 2011; Strouse et al. 1998). However, older adults can overcome auditory processing difficulties by utilizing supportive strategies, such as internally stored knowledge or acoustic cues, to compensate for cognitive and auditory processing challenges (Pichora‐Fuller 2008). In this context, VR offers valuable opportunities to investigate how aging and other individual factors influence the neural mechanisms underlying cognitive and auditory processing in real‐world‐like environments. It facilitates insights that traditional experimental setups may not easily provide (Parsons 2015). Nonetheless, it is important to evaluate the ecological validity of virtual environments and ensure that findings are comparable to those obtained in real‐world settings or traditional laboratory conditions.

To assess the validity of virtual environments, neurophysiological measures can offer valuable insights into the neurocognitive processes involved during tasks—insights that may not be fully captured by behavioral performance or subjective evaluations alone (Choi et al. 2023). For instance, Kalantari et al. (2021) found no significant differences in electroencephalogram (EEG) frequency band‐power and event‐related potentials (ERPs) between real and virtual classroom settings during cognitive tasks, suggesting a high validity of the VR setting. ERPs, which are time‐locked electrical responses measured from the continuous EEG, reflect the brain's activity in response to specific sensory, cognitive, or motor events. They have been used, for example, to examine how younger and older adults perceive and process spatial changes in an auditory environment (e.g., Lewald and Getzmann 2015). Thus, ERPs are valuable for examining both task‐specific and individual‐specific influences on auditory processing.

Early stimulus processing is reflected by the P1, N1, and P2 potentials. Whereas the P1 primarily reflects the physical characteristics of auditory stimuli and is more related to sensory processing than cognitive or attentional factors (Eggermont and Ponton 2002; Grunwald et al. 2003), the N1 signifies the initial stages of attentional focus and orientation toward novel stimuli (Näätänen and Picton 1987). Besides, the P2 reflects more complex cognitive processes, such as stimulus classification and relevance evaluation (Arnott et al. 2011).

Auditory change detection tasks, or oddball paradigms, are effective in eliciting a series of deviance‐related auditory evoked potentials (Duncan et al. 2009; Korka et al. 2022; Polich 2007). In these tasks, participants either listen passively to a sequence of stimuli or actively focus on detecting a deviant stimulus among standard stimuli. In both conditions, deviant/target stimuli evoke ERPs that are associated with the perception of auditory changes, including the mismatch negativity (MMN) and the subsequent P3. These components are derived by subtracting the averaged ERP evoked by the standard sound from that evoked by the deviant/target sound. The MMN reflects preattentive processing and deviation detection (Escera and Corral 2007; Näätänen et al. 2007; Rutiku et al. 2024), whereas the P3 is primarily associated with stimulus processing and evaluation (Katayama and Polich 1998; Polich 2007; Rutiku et al. 2024). The MMN increases with physical difference between standard and deviant stimuli (Berti et al. 2004) and can be triggered by changes in intensity, duration, frequency, or pitch (Paavilainen 2013), which, however, also applies to the N1 component, which can overlap with the MMN and may contribute to the interpretation of MMN amplitude variations in some studies (Horváth et al. 2008). Additionally, the MMN can be elicited by changes in sound location, whether delivered via loudspeakers (Deouell et al. 2006; Lewald et al. 2018; Paavilainen et al. 1989) or headphones (Altman et al. 2005; Matthews et al. 2007). According to Sonnadara et al. (2006), occasional changes in sound location elicit an early MMN, suggesting an early‐warning system for new sound sources. In contrast, the P3's subcomponent, P3b, is closely linked to context updating and the processing of task‐relevant stimuli (Polich 2007; Verleger et al. 2005), serving as a marker of cognitive resource allocation (Getzmann et al. 2022; Katayama and Polich 1998). The amplitude and latency of the P3b are influenced by factors such as the difficulty of target discrimination (Comerchero and Polich 1999; Lewald et al. 2018) and mental workload (Kok 2001).

It has already been demonstrated that age can affect ERP characteristics, offering insights into the functional integrity of auditory processing across the lifespan, such as declines in both sensory and cognitive dimensions (X. Wang et al. 2024). For example, in a study by Getzmann et al. (2015), no differences in the amplitude of the P1 and P2 were found between younger and older adults. However, older participants showed a larger N1 amplitude compared with younger participants during a speech perception task. This finding suggests that older adults may engage in stronger early attentional processes, potentially due to compensatory mechanisms that enhance specific cognitive functions. Additionally, no age‐related effects were observed on the latency of the P1 and N1 components. However, older adults showed a delayed P2 component, with increased activity in prefrontal areas, which may also indicate functional compensation. Further, older adults exhibit a reduced MMN response, particularly at longer interstimulus intervals, suggesting a decline of sensory memory with age (Cheng et al. 2013; Cooper et al. 2006; Ruzzoli et al. 2012). In addition, an age‐related increase in MMN peak latency was observed, also indicating lower auditory discrimination accuracy in older adults, possibly related to cognitive decline (Näätänen et al. 2012). Additionally, Chow et al. (2025) showed that MMN amplitudes attenuate with increasing age and could serve as an indicator of mnemonic discrimination, which in turn provides insights into how age‐related memory impairment is related to encoding differences between age groups. Aging is further associated with distinct changes in the P3b component, with research showing a linear decline in its amplitude over time (Fjell and Walhovd 2004; O'Connell et al. 2012). Although older adults exhibit greater variability in reaction times, the latency of the P3b remains relatively stable. This indicates age‐related modifications in neural activation and cognitive processing (Fjell et al. 2009).

In this study, we tested the validity of a virtual test environment for the investigation of auditory spatial attention across younger and older adults. Therefore, we conducted two consecutive experiments: one in a real and the other in a virtual environment. The participants were instructed to respond to sound signals whenever their position changed in azimuth or distance relative to a central reference position. In the real environment, sound stimuli were played back through physical loudspeakers, whereas in the virtual environment, sounds were binaurally rendered and delivered via headphones. Additionally, participants wore a head‐mounted display (HMD) that presented a virtual replica of the real laboratory room. To assess performance in both environments and evaluate the validity of the virtual test environment for neurocognitive research, we collected both behavioral and neurophysiological data (EEG). Here, we focused on ERPs associated with fundamental auditory processing, including components related to sound detection (P1‐N1‐P2 complex), preattentive deviance detection and discrimination (MMN), and target processing (P3b). Since all these components are sensitive to the characteristics of auditory cues (such as frequency, intensity, and temporal changes), they can serve as objective measures for comparing different environments in terms of how consistently or inconsistently auditory stimuli are processed.

We hypothesized that if the virtual environment accurately replicates the real environment, we should observe comparable task performance (i.e., accuracy in detecting target sounds) and similar neurophysiological responses, as indicated by ERPs, in both environments. Additionally, we anticipated improved target detection for sounds changed in azimuth positions compared with those varying in egocentric distance from the listener, expecting similar effects in both environments. Building on previous findings regarding the impact of age on sound discrimination and the underlying neural processes, we aimed to replicate these results while observing age‐related differences that are independent of the environment. In particular, we expected a more pronounced N1, but decreased MMN and P3 in the older compared with the young group, whether in the real or virtual environment.

## 2 Materials and Methods

### 2.1 Participants

A total of 44 participants took part in this experiment. Of these, 22 participants (10 female, 12 male) aged between 20 and 31 years (*M* = 24.00, *SD* = 3.19) were assigned to the younger age group, whereas 22 participants (13 female, 9 male) aged between 55 and 70 years (*M* = 64.77, *SD* = 4.47) were assigned to the older age group. All participants reported no neurological diseases, being free of medication, as well as normal vision and hearing. Furthermore, all participants were right‐handed. The experiment took place on two different testing days, whereas the starting environment (real or virtual) was counterbalanced. Participants usually had a 14‐day interval between their two testing sessions. Participants received written instructions for the upcoming task on each testing day.

**FIGURE 1:**
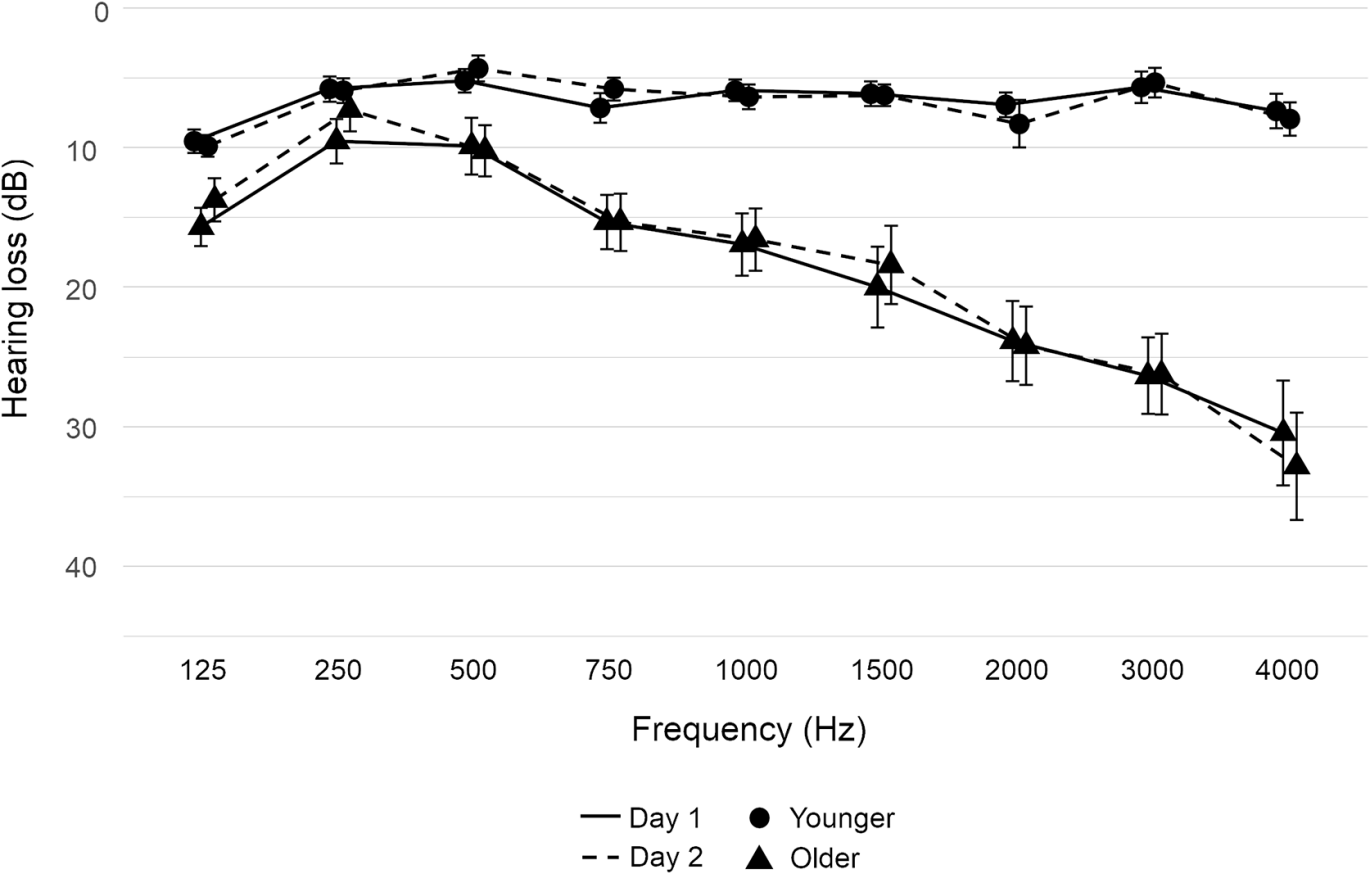
Pure‐tone audiograms of younger and older participants on both testing days. Error bars represent standard deviations.

Prior to the experiment on both testing days, all participants performed a pure‐tone audiometry using frequencies between 125 Hz and 4000 Hz (Oscilla USB 330; Inmedico, Lystrup, Denmark). On both days, hearing thresholds of both age groups were within an acceptable range (< 35‐dB hearing loss at frequencies ≤ 4000 Hz). A repeated measures ANOVA with age (younger, older) as between‐subject factor and frequency (125, 250, 500, 750, 1000, 1500, 2000, 3000, and 4000 Hz) as well as testing day (Day 1, Day 2) as within‐subject factors revealed main effects of age (*F*_1,42_ = 35.19, *p* < 0.001, *ƞ*_*p*_^2^ = 0.546) and frequency (*F*_3.50,146.99_ = 24.43, *p* < 0.001, *ƞ*_*p*_^2^ = 0.368), as well as an interaction effect of age and frequency (*F*_8,336_ = 20.13, *p* < 0.001, *ƞ*_*p*_^2^ = 0.324). These results indicate higher hearing loss in older participants, especially in the higher frequency range, whereas no differences were observed between the two testing days (see Figure 1).

The study adhered to the ethical guidelines outlined by the Code of Ethics of the World Medical Association (Declaration of Helsinki) and received approval from the local Ethics Committee of the Leibniz Research Centre for Working Environment and Human Factors in Dortmund, Germany. All individuals provided written informed consent prior to the experiment and were paid 12€ per hour or received equivalent study credits.

### 2.2 Real Environment

**FIGURE 2:**
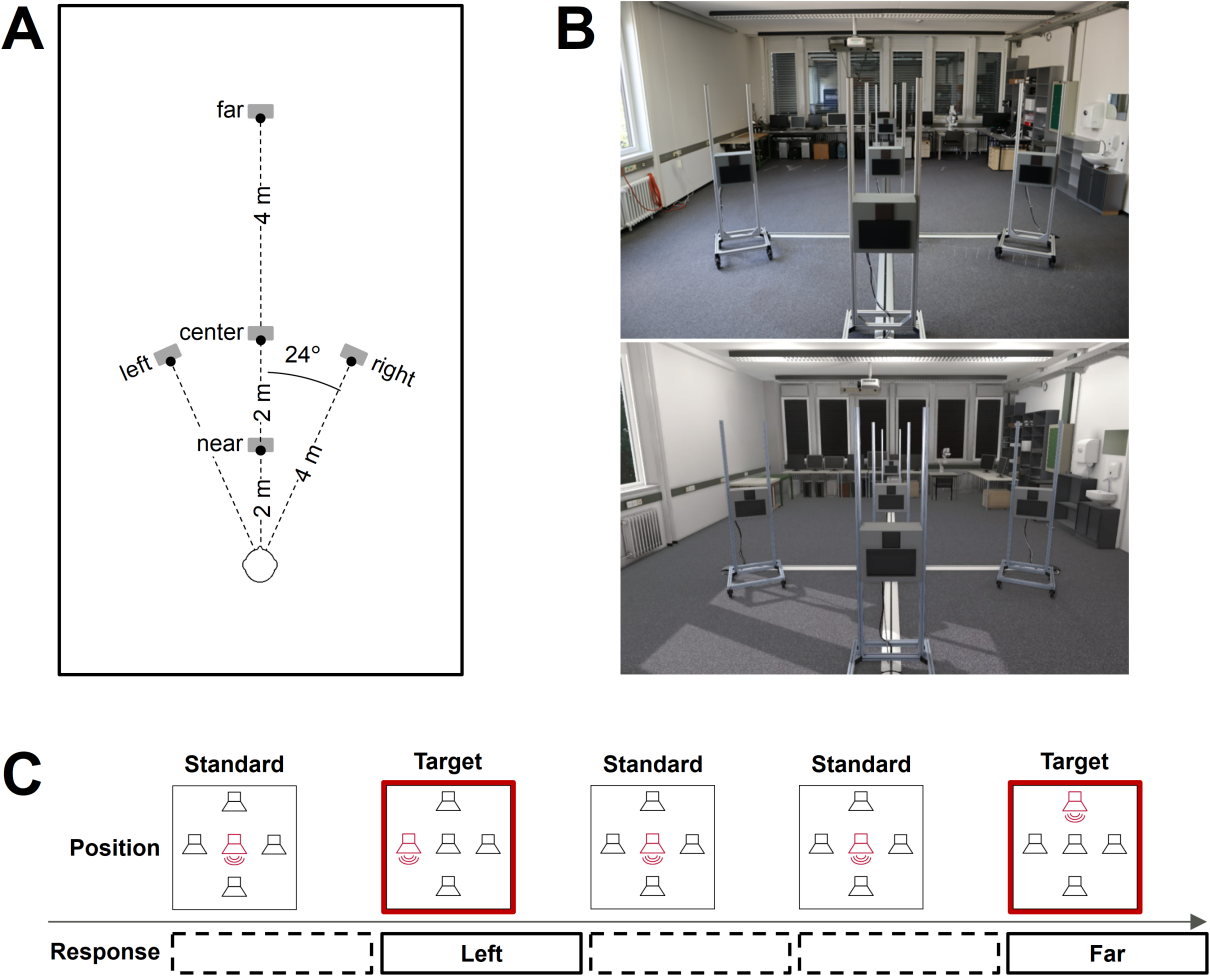
(A) Top view of the participant sitting in front of five loudspeakers positioned at different locations in the room. (B) Photos of the real laboratory environment from the participant's perspective (top) and the virtual environment presented via HMD (bottom). (C) Exemplary sequence of the auditory change detection task. Participants had to respond via joystick whenever the sound position changed from the standard (center) loudspeaker to any target loudspeaker (left, right, near, or far).

The experimental room was the same as described in Stodt et al. (2024). It consists mainly of concrete walls, a carpeted floor, and large windows on the left and rear sides. Its dimensions are 7.22 m (width) × 12 m (length) × 3.4 m (height), resulting in an area of 87 m^2^ and a volume of 294 m^3^. All in all, the room has a broadband reverberation time *T*_60_ ≈ 0.8 s, resulting in a critical distance *d*_*c*_ ≈ 3.5 m and a Schroeder frequency *f*_*S*_ ≈ 104 Hz. Participants were seated on a height‐adjustable chair in the middle between the left and the right wall and 2 m away from the back wall. Five loudspeakers (Visaton SC 8 N, 8 Ohm, 8 cm) were placed in front of the participants: Three loudspeakers on the median plane at distances of 2 m (near), 4 m (center), and 8 m (far), as well as two loudspeakers on the left and right side (*θ* = ±24°) of the center loudspeaker (see Figure 2A). Each loudspeaker was mounted in a wooden box and placed at a height of *h*_*LS*_ = 1.14 m. The wooden box also included a small screen for visual stimulus presentation, although it was not used in this experiment. Participants were positioned at an ear height of *h*_*P*_ = 1.8 m and frontal gaze direction, which was additionally ensured by using a chinrest during the experiment. This resulted in elevation differences of −18.2° (for the near loudspeaker), −9.4° (for the center, left, and right loudspeaker), and −4.7° (for the far loudspeaker) relative to the participant's ears.

### 2.3 Virtual Environment

The virtual environment was presented to the participants while wearing an HMD (HTC Vive Pro 2) at the same sitting position as in the real environment. Blender was used for modeling the visual modality of the laboratory room according to its real dimensions including all objects and furniture with realistic textures. Unity was used to render the scene and for experimental control (Bebko and Troje 2020). A direct comparison of the real and virtual environments is shown in Figure 2B.

For audio playback, we employed external headphones (Sennheiser HD 25 Plus) with a compensation filter using the approach of Masiero and Fels (2011). Virtual sounds were created by convolving the original sound stimulus with the BRIRs. These were recorded for each of the five loudspeaker positions with in‐ear microphones (Brüel & Kjær type 4101‐B) mounted to a dummy head (Head Acoustics HMS II.3–33 head‐and‐torso simulator [HATS]) at the sitting position of the participants. Those BRIRs include all information about sound propagation from a loudspeaker placed at a specific position in the room to the receiving participant (including reflections, absorption, and timbre) and therefore enable a plausible reproduction of the real room acoustics (Blauert 1996; Møller 1992; Zhang et al. 2017).

### 2.4 Stimulus, Task, and Procedure

In an auditory change detection (oddball) task, sequences of a 500‐ms pink noise burst (10‐ms fade‐in, 10‐ms fade‐out, band‐pass filtered to a frequency range of 125 Hz to 4 kHz) were presented to the participants. Each stimulus was followed by an interstimulus interval of 1300–1700 ms. In 684 out of 900 total trials (76%), the stimulus was emitted from the center loudspeaker position, which acted as the standard. In the remaining trials, the stimulus was randomly emitted by one of the four target positions: right, left, near, or far, with each target position occurring in 54 trials (6% of the overall trials). After each target, a sequence of at least two and at most six standards was presented again. Participants were instructed to react whenever the sound position shifted from the center loudspeaker to any target loudspeaker, using a 2‐axis joystick to indicate the target position (left target ≙ joystick to the left, right target ≙ joystick to the right, near target ≙ pulling the joystick toward oneself, far target ≙ pushing the joystick away from oneself). For the standard (center loudspeaker), participants were instructed not to provide any input (see Figure 2C). Participants were informed that they did not need to react immediately when a target occurred and should wait until the sound faded. After every 300 trials, participants were given the opportunity to take a short break. The rate of correctly allocated targets per loudspeaker position, environment, and age group was determined. Correct responses were averaged for the left and right targets (azimuth dimension) and for the near and far targets (distance dimension). To correct for variance heterogeneity, accuracy data were logit‐transformed for subsequent analyses.

To ensure precise timing of sound onsets at participants' ears regardless of the loudspeaker position, we measured and compensated for the time of arrival of the stimulus from different positions in the real environment.

### 2.5 EEG Recording and ERP Latency Alignment

Throughout the entire experiment, continuous EEG signals were recorded using 64 Ag/AgCl electrodes (EasyCap, Wörthsee, Germany) based on the international 10–20 system, with a sampling rate of 1000 Hz (QuickAmp DC Amplifier, BrainVision, Gilching, Germany). Electrode impedances were kept below 10 kΩ. During recording, AFz served as the ground electrode and FCz as the reference electrode. The HMD was placed directly on the EEG cap, with slim custom‐made distancers positioned under the head strap to prevent it from contacting the central midline electrodes.

In the technical implementation of the experiment in both the real and virtual environments, we ensured that the onsets of sound stimuli were accurately synchronized with the EEG data. This involved making necessary adjustments for sound travel times, as previously described. Despite these efforts, small latency differences between the sound onset markers and the actual sound onset at the participant's position can arise because of the varied software, hardware, and implementation methods used in both environments. These latency differences in the sound onset can potentially affect the resulting ERPs, especially the difference waveforms. To address this, we decided to use the auditory P1 component, which is a prominent marker reflecting the early processing of sensory input (Alain and Tremblay 2007) and aligned all ERPs to the peak latency of the P1. This alignment ensures that the characteristics of the later ERPs of interest, such as the MMN and P3b, are not influenced by technical latency differences, allowing an accurate interpretation of the underlying neurocognitive processes. For this, we first preprocessed all EEG data as described later in Section 2.6. We then examined the latency of the P1 component in the ERP averaged over all stimulus positions (standards, targets), environments (real, virtual), and age groups (young, old), including only trials with correct responses. The averaged P1 had a peak latency of 57 ms derived at a grid of six frontocentral electrodes (Fz, F1, F2, FCz, FC1, FC2). We then calculated the P1 peak latency at the same electrode positions for each combination of environment, age group, and sound position. Each sound onset marker was then adjusted by the difference between its respective latency and the 57‐ms peak latency of the P1 component. In the real environment, the average shift was −4.10 ms (SD = 8.12 ms), with a minimum of −19 ms and a maximum of 6 ms. In the virtual environment, the mean shift was 4.60 ms (SD = 4.56 ms), with a minimum of −2 ms and a maximum of 11 ms. Finally, we preprocessed the raw EEG data again with the adjusted sound onsets.

### 2.6 EEG Preprocessing and Analysis

EEG data were preprocessed offline using the EEGLAB (v2024.0; Delorme and Makeig 2004) and ERPLAB (v10.04; Lopez‐Calderon and Luck 2014) toolboxes in MATLAB, along with custom code, separately for each participant and environment. A Hamming‐windowed sinc FIR high‐pass filter (cutoff: 0.25 Hz, filter length: 6601, transition bandwidth: 0.5 Hz) and low‐pass filter (cutoff: 33.75 Hz, filter length: 441, transition bandwidth: 7.5 Hz) were applied to the raw data. Channels corresponding to defective electrodes (e.g., those with excessive noise or flat lines) were removed and interpolated using spherical interpolation. The data were then re‐referenced to the average signal of all 64 electrodes. Further data cleaning was performed using independent component analysis (ICA). For this, a subset of the EEG data was generated with cut‐off frequencies of 1 and 30 Hz, down‐sampled to 200 Hz for faster computation, and only every second trial included. The continuous EEG signal was then segmented into 1200‐ms stimulus‐locked epochs, covering the period from −200 to 1000 ms relative to stimulus onset. The interval from −200 ms to stimulus onset served as the prestimulus baseline. Here, only epochs in which participants responded correctly were included (i.e., a correct response after a target and no response after a standard). Additionally, each first standard following a target was excluded (216 trials per participant) to eliminate possible effects of post‐deviance distraction (Getzmann and Wascher 2016). After applying an automatic artifact rejection (resulting in *M*_real_ = 23.75, *SD*_real_ = 15.60 removed epochs per participant in the real environment; *M*_virtual_ = 22.59, *SD*_virtual_ = 15.26 removed epochs in the virtual environment; each in the preprocessing pipeline with adjusted sound onsets), the ICA was computed. The ICLabel algorithm (v1.6; Pion‐Tonachini et al. 2019) was then applied, and the obtained results and IC weights were transferred to the original EEG data. Components with a probability of less than 30% for the brain category or more than 30% for artifactual categories such as eye, muscle, or heart activity were excluded (*M*_real_ = 28.70, *SD*_real_ = 6.98 removed components per participant in the real environment; *M*_virtual_ = 30.00, *SD*_virtual_ = 7.54 removed components in the virtual environment). By applying amplitude thresholds, the remaining artifactual and noisy epochs were subsequently removed (*M*_real_ = 1.93, *SD*_real_ = 4.34 removed epochs per participant in the real environment; *M*_virtual_ = 2.00, *SD*_virtual_ = 4.49 removed epochs in the virtual environment; each in the preprocessing pipeline with adjusted sound onsets).

Subsequently, all standard epochs were averaged separately for each environment and age group. The remaining target epochs were averaged per environment and age group based on their spatial dimensions: Epochs originating from left and right targets (azimuth dimension) were averaged, as were those from near and far targets (distance dimension). After excluding incorrect trials, the first standards after each target, and rejecting artefactual epochs, the standard ERPs were based on an average of *M*_standard,real_ = 452.96 (*SD*_standard,real_ = 57.59) standard stimuli per participant for the real environment, and *M*_standard,virtual_ = 458.98 (*SD*_standard,virtual_ = 33.31) for the virtual environment. The target ERPs were based on an average of *M*_azimuth,real_ = 104.55 (*SD*_azimuth,real_ = 5.19) azimuth targets, and *M*_distance,real_ = 102.43 (*SD*_distance,real_ = 9.37) distance targets per participant for the real environment. For the virtual environment, the average number of included targets was *M*_azimuth,virtual_ = 102.91 (*SD*_azimuth,virtual_ = 7.34) azimuth targets, and *M*_distance,virtual_ = 99.55 (*SD*_distance,virtual_ = 11.12) distance targets.

For quantifying the characteristics of the ERPs of interest (amplitudes and latencies), we utilized the MATLAB function by Liesefeld (2018). This function also allowed us to implement a jackknifing procedure to address potential excessive noise in data from individual participants, which could otherwise distort the averaged ERP measures. In this approach, we calculated *n* averaged scores (such as amplitude or latency of an ERP), where each score was obtained by averaging across *n* − 1 participants. This method resulted in a dataset of *n* scores, where each score represented the grand average of all *n* − 1 individual scores (Hansen and Hillyard 1980; Miller et al. 2009; Smulders 2010; Ulrich and Miller 2001). The jackknifing procedure effectively reduces noise in the dataset without the need to exclude individual data or lose interindividual variance, while simultaneously not affecting the ERP waveforms themselves (Wascher et al. 2022).

Based on a visual inspection of the topographies for the time windows corresponding to the expected ERP components, as well as the scalp distribution of negative and positive polarities and areas highlighted in the relevant literature, specific electrodes were selected for further analyses. To quantify the early P1, N1, and P2 components for each participant in both environments, we measured the peak amplitude of each component within a predefined time window corresponding to its expected occurrence (between 50 and 60 ms for the P1, between 50 and 200 ms for the N1, and between 100 and 400 ms for the P2). To derive the MMN and P3b, difference waveforms were calculated by subtracting the averaged standard ERPs from the corresponding averaged dimension‐related target ERPs for each participant per environment. For quantification of both difference potentials, we used the mean amplitude as well as the fractional area latency (FAL; Hansen and Hillyard 1980; Kiesel et al. 2008). The FAL is defined as the timepoint where the ERP waveform reaches a specific percentage of its total area (50% in our study). This measure is advantageous as it is not significantly affected by high‐frequency noise in the EEG signal or by a low number of trials contributing to the averaged ERP waveforms. These factors can sometimes cause multiple local maxima in the temporal search window, where the highest peak may not represent the center or true representation of the ERP component (Wascher et al. 2022). A time window of 20 ms around the individual FAL was used to compute the mean amplitudes of the MMN and P3b.

Resulting amplitudes and latencies were subsequently used for testing the statistical significance by using repeated measures ANOVAs with the between‐subjects factor age group (younger vs. older), the within‐subjects factors environment (real vs. virtual), and target dimension (azimuth vs. distance), as well as post hoc *t*‐tests for the potentially found interaction effects. Post hoc *t*‐tests were corrected for multiple testing using false discovery rate correction (Benjamini and Hochberg 1995).

To test our hypothesis that neurophysiological responses to changes in auditory spatial positions are comparable and not significantly different between real and virtual environments, we also performed null hypothesis significance testing with Bayes factor (BF) analyses (Kass and Raftery 1995). These allowed us to assess whether nonsignificant results supported our null hypotheses or if the data were simply insensitive (see Dienes 2014; Morey and Rouder 2011; Rouder et al. 2009). BFs quantify how much more likely the data are under the alternative (H_1_) than the null hypothesis (H_0_) (Kass and Raftery 1995). A BF > 1 favors H_1_, a BF < 1 favors H_0_, and a BF = 1 indicates no preference. Jeffreys (1961) proposed cut‐offs categorizing evidence from anecdotal to extreme. In our study, we performed BF paired one‐sample *t*‐tests using the BayesFactor package in R (v0.9.12.4.7; Morey and Rouder 2022) with default Cauchy priors (Greber et al. 2018). All statistical analyses were carried out using R.

## 3 Results

### 3.1 Detection Accuracy

**FIGURE 3:**
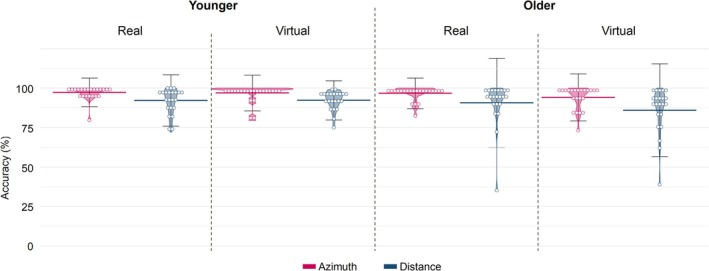
Accuracy in detecting the targets in azimuth and distance, shown separately for both environments and age groups. Horizontal bold lines represent the mean values of performance over all participants, vertical bars represent ± one standard deviation, and dots indicate the individual mean accuracy values.

Figure 3 shows the mean accuracy in detecting target sounds in azimuth and distance per age group and environment. Repeated measures ANOVAs revealed a significant main effect of target dimension (*F*_1,42_ = 77.82, *p* < 0.001, *ƞ*_*p*_^2^ = 0.649), indicating that participants were more accurate in detecting targets in azimuth compared with distance (*F*_1,42_ = 77.82, *p* ≤ 0.001, *ƞ*_*p*_^2^ = 0.649). Furthermore, a small trend indicating a higher accuracy in the real environment compared with the virtual environment could be observed (*F*_1,42_ = 3.32, *p* = 0.076, *ƞ*_*p*_^2^ = 0.073). No further significant main effects nor any interaction effects were observed (all *F* ≤ 1.84, all *p* ≥ 0.182, all *ƞ*_*p*_^2^ ≤ 0.042).

### 3.2 ERP Analyses

#### 3.2.1 Topographies

**FIGURE 4:**
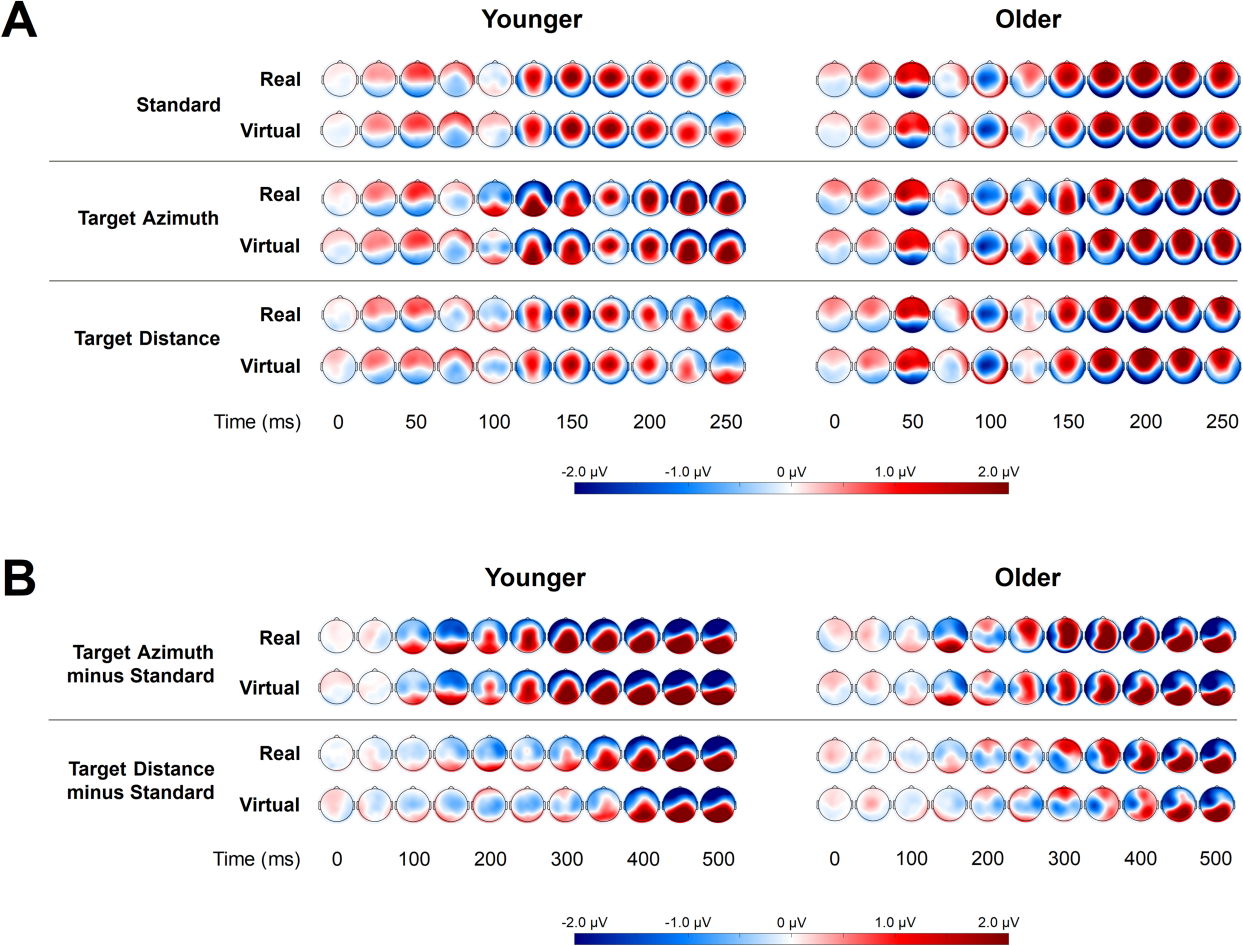
(A) Averaged EEG topographies for the first 250 ms after the onset of the standard stimulus (covering the early P1, N1, and P2 potentials) as well as the azimuth and distance targets. Each time series is shown for both age groups and environments. (B) Averaged topographies of the difference ERPs for the first 500 ms after stimulus onset (covering the MMN and P3b). Each time series is shown for both age groups and environment.

The standard stimulus and the target stimuli (summarized in the azimuth or distance dimension) evoked a prominent frontocentral P1‐N1‐P2 complex within the first 250 ms (see Figure 4A). Therefore, data were averaged across six frontocentral electrodes (Fz, F1, F2, FCz, FC1, and FC2) to analyze the ERP characteristics.

Figure 4B illustrates the topographies of the difference ERPs (targets minus standard) within the first 500 ms after stimulus onset. From approximately 100 ms onward, a negativity (respectively in this case the MMN) expands in the frontal area, with a potentially more intense amplification to the right frontal hemisphere. To analyze and illustrate the MMN, data were averaged across a broad grid of electrodes, covering an area from the anterior right hemisphere to the frontocentral midline (AF4, AF8, Fz, F2, F4, F6, FCz, FC2, FC4, FT8, Cz, C2, C4, C6, and T8). From approximately 250 ms onward (depending on the target dimension), a strong positivity, the P3b, appears in the parietal areas. Accordingly, we chose a central parietal grid of electrodes (CP1, CPz, CP2, P1, Pz, and P2) where the EEG signal was averaged and used for the upcoming analyses of the P3b characteristics.

An initial visual inspection did not indicate substantial qualitative differences in the ERPs in the real and virtual environments, both for the P1‐N1‐P2 complex and the difference ERPs and for the young and older participants. In the following, effects of age, environment, and target dimension, as well as their interaction effects on the peak amplitude of the P1, N1, and P2, as well as on the mean amplitudes and area latencies of the MMN and P3b components, are described.

#### 3.2.2 P1‐N1‐P2

**FIGURE 5:**
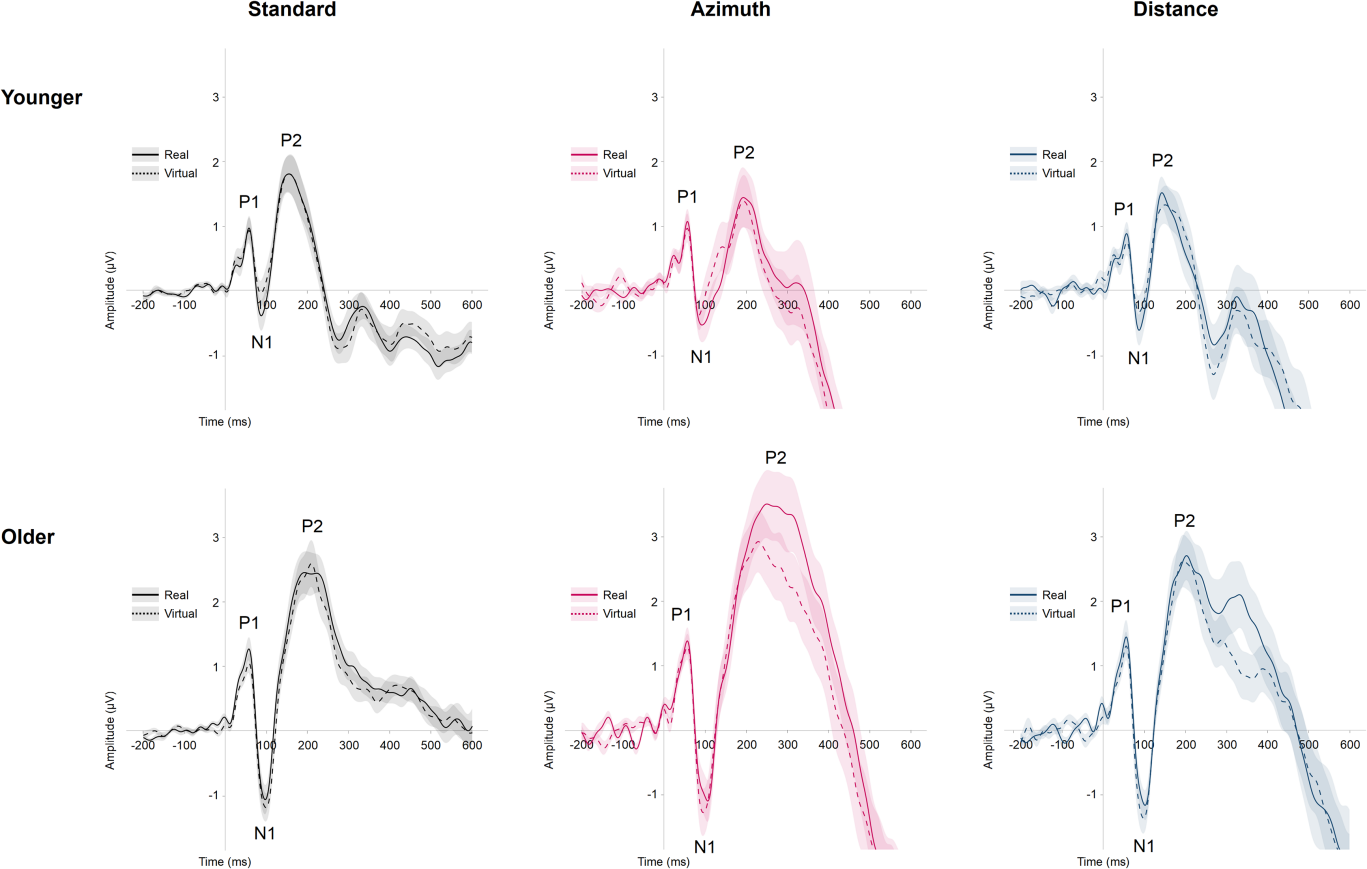
Grand averaged ERP waveforms from standard and target trials at electrodes Fz, F1, F2, FCz, FC1, and FC2, comparing the P1‐N1‐P2 complex in the real (solid lines) and virtual environment (dashed lines) for both age groups. Shaded areas beyond the waveforms refer to the range of ± one standard error.

Figure 5 provides a visual comparison of the P1‐N1‐P2 complex between the real and virtual environments for each target dimension and age group. For the amplitude of the P1, there was a marginal effect of age (*M*_younger_ = 0.88 μV vs. *M*_older_ = 1.32 μV, *F*_1,42_ = 3.06, *p* = 0.088, *ƞ*_*p*_^2^ = 0.068), suggesting a potential trend where older participants tend to show a higher P1 amplitude than younger participants, regardless of the environment and target dimension. No main effects of environment or target dimension nor any interaction effects were found (all *F* ≤ 1.86, all *p* ≥ 0.180, all *ƞ*_*p*_^2^ ≤ 0.042).

The amplitude of the N1 was more pronounced in the older compared with the younger group (*M*_younger_ = −0.45 μV vs. *M*_older_ = −1.20 μV, *F*_1,42_ = 4.82, *p* = 0.034, *ƞ*_*p*_^2^ = 0.103). The age‐related difference was more pronounced in the virtual environment (*M*_diff,virtual_ = −0.96 μV) compared with the real environment (*M*_diff,real_ = −0.54 μV). Post hoc *t*‐test showed a significant difference between environments (*T* = 2.18, *p* = 0.034), with a mean difference of *M* = 0.22 μV for the younger group and *M* = −0.20 μV for the older group. This pattern was reflected in a significant interaction between age and environment (*F*_1,42_ = 4.76, *p* = 0.035, *ƞ*_*p*_^2^ = 0.102). No further main or interaction effects on the amplitude of the N1 were found (all *F* ≤ 0.16, all *p* ≥ 0.692, all *ƞ*_*p*_^2^ ≤ 0.004).

There was a significant main effect of age on the amplitude of the P2, with a higher amplitude in the older group compared with the younger group (*M*_younger_ = 1.41 μV vs. *M*_older_ = 2.94 μV, *F*_1,42_ = 9.20, *p* = 0.004, *ƞ*_*p*_^2^ = 0.180). Furthermore, there was a marginal effect of environment on P2 amplitude, with the real environment eliciting a slightly higher P2 amplitude than the virtual environment (*M*_real_ = 2.29 μV vs. *M*_virtual_ = 2.06 μV, *F*_1,42_ = 3.79, *p* = 0.058, *ƞ*_*p*_^2^ = 0.083). No further main or interaction effects on the P2 amplitude were found (all *F* ≤ 1.67, all *p* ≥ 0.204, all *ƞ*_*p*_^2^ ≤ 0.038).

#### 3.2.3 MMN

Regarding the MMN amplitude, there were significant main effects of age (*F*_1,42_ = 4.50, *p* = 0.040, *ƞ*_*p*_^2^ = 0.097), environment (*F*_1,42_ = 13.97, *p* = 0.001, *ƞ*_*p*_^2^ = 0.250), and target dimension (*F*_1,42_ = 49.24, *p* < 0.001, *ƞ*_*p*_^2^ = 0.540). The MMN was more pronounced in the younger compared with the older group (*M*_younger_ = −0.82 μV vs. *M*_older_ = −0.49 μV), in the real compared with the virtual environment (*M*_real_ = −0.80 μV vs. *M*_virtual_ = −0.51 μV), and for the azimuth compared with the distance targets (*M*_azimuth_ = −0.98 μV vs. *M*_distance_ = −0.33 μV). No interaction effects on the MMN mean amplitude were observed (all *F* ≤ 1.97, all *p* ≥ 0.168, all *ƞ*_*p*_^2^ ≤ 0.045).

For the FAL of the MMN, there were significant main effects of environment (*F*_1,42_ = 5.73, *p* = 0.021, *ƞ*_*p*_^2^ = 0.120) and target dimension (*F*_1,42_ = 7.77, *p* = 0.008, *ƞ*_*p*_^2^ = 0.156). The MMN occurred slightly later in the virtual compared with the real environment (*M*_virtual_ = 180.73 ms vs. *M*_real_ = 156.70 ms). In addition, targets on the distance dimension evoked a delayed MMN compared with azimuth targets (*M*_distance_ = 189.61 ms vs. *M*_azimuth_ = 147.82 ms). Beyond that, there was a marginal interaction effect between environment and target dimension (*F*_1,42_ = 2.93, *p* = 0.094, *ƞ*_*p*_^2^ = 0.065). No further main or interaction effects were observed (all *F* ≤ 1.84, all *p* ≥ 0.182, all *ƞ*_*p*_^2^ ≤ 0.042).

**FIGURE 6:**
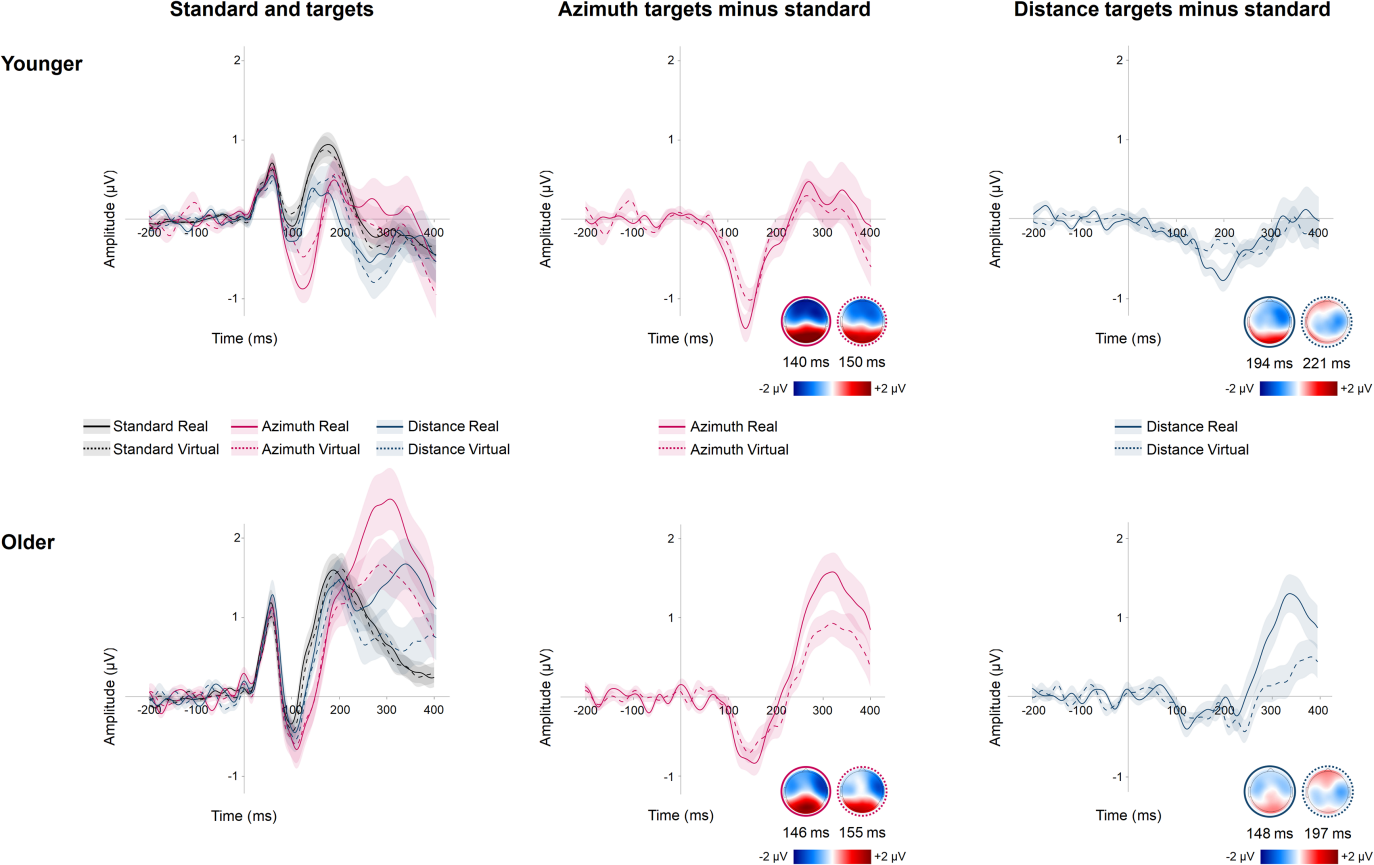
Grand‐averaged ERP waveforms recorded in the real (solid lines) and virtual (dashed lines) environments for both age groups at electrodes AF4, AF8, Fz, F2, F4, F6, FCz, FC2, FC4, FT8, Cz, C2, C4, C6, and T8. Left panel: ERPs elicited by standard and target stimuli. Middle and right panels: ERP difference waveforms (target minus standard) showing the MMN. Shaded areas beyond the waveforms refer to the range of ± one standard error. Topographic maps refer to the point in time when the area of the respective component reaches 50% (FAL).

Figure 6 illustrates the comparison of the MMN between the real and virtual environments, separated by each target dimension and age group. Topographic maps show the distribution of negative and positive polarities on the scalp at the timepoint when the respective component reaches 50% of its area size (FAL).

#### 3.2.4 P3b

For the mean amplitude of the P3b, significant main effects of environment (*F*_1,42_ = 10.99, *p* = 0.002, *ƞ*_*p*_^2^ = 0.207) and target dimension (*F*_1,42_ = 28.67, *p* < 0.001, *ƞ*_*p*_^2^ = 0.406) were found. The P3b was more pronounced in the real environment compared with the virtual environment (*M*_real_ = 3.17 μV vs. *M*_virtual_ = 2.56 μV), as well as for the azimuth compared with distance targets (*M*_azimuth_ = 3.28 μV vs. *M*_distance_ = 2.45 μV). Beyond that, a marginal interaction effect was observed between age and environment (*F*_1,42_ = 3.01, *p* = 0.090, *ƞ*_*p*_^2^ = 0.067). Here, the difference in P3b amplitude between the real and virtual environments was descriptively more pronounced in older than in younger participants (*M*_diff,older_ = 0.92 μV vs. *M*_diff,younger_ = 0.29 μV, *T* = 1.73, *p* = 0.090). Furthermore, a trend toward a three‐way interaction effect between age, environment, and target dimension was observed (*F*_1,42_ = 2.91, *p* = 0.095, *ƞ*_*p*_^2^ = 0.065). Post hoc analyses revealed that P3b amplitudes for both target dimensions were equivalent between the real and virtual environments in younger participants and also between younger and older participants within the real environment. In contrast, older participants exhibited significantly lower P3b amplitudes in the virtual environment compared with the real environment for the azimuth dimension (*M*_older,real,azimuth_ = 3.62 μV vs. *M*_older,virtual,azimuth_ = 2.54 μV, *T* = 3.59, *p* = 0.009), as well as for the distance dimension (*M*_older,real,distance_ = 2.79 μV vs. *M*_older,virtual,distance_ = 2.03 μV, *T* = 3.54, *p* = 0.009). No further main or interaction effects were found (all *F* ≤ 1.02, all *p* ≥ 0.317, all *ƞ*_*p*_^2^ ≤ 0.024).

Regarding the FAL of the P3b, there were significant main effects of the environment (*F*_1,42_ = 11.23, *p* = 0.002, *ƞ*_*p*_^2^ = 0.211) and target dimension (*F*_1,42_ = 211.55, *p* < 0.001, *ƞ*_*p*_^2^ = 0.834), indicating a delayed P3b in the virtual compared with the real environment (*M*_virtual_ = 530.01 ms vs. *M*_real_ = 495.23 ms), as well as for the distance targets compared with the azimuth targets (*M*_distance_ = 575.45 ms vs. *M*_azimuth_ = 449.78 ms). Furthermore, there was a significant interaction effect between age group and target dimension (*F*_1,42_ = 5.26, *p* = 0.027, *ƞ*_*p*_^2^ = 0.111). The age‐related difference in P3b latency was more pronounced for the distance dimension (*M*_diff,distance_ = 25.82 ms) compared with the azimuth dimension (*M*_diff,azimuth_ = −13.80 ms). A post hoc test revealed a significant difference between target dimensions (*T* = 2.29, *p* = 0.032), with a mean difference of *M* = 145.48 ms for the younger group and *M* = 105.86 ms for the older group.

Moreover, a significant interaction effect was observed between the environment and target dimension (*F*_1,42_ = 7.70, *p* = 0.008, *ƞ*_*p*_^2^ = 0.155). Specifically, the difference in P3b latency between environments was more pronounced for the distance targets (*M*_diff,distance_ = 50.59 ms) than for azimuth targets (*M*_diff,azimuth_ = 18.98 ms). However, with a mean difference of *M* = 141.48 ms for the virtual environment and *M* = 109.86 ms for the real environment, a post hoc test revealed no significant differences between environments (*T* = 0.78, *p* = 0.441). No further main or interaction effects were found (all *F* ≤ 0.41, all *p* ≥ 0.524, all *ƞ*_*p*_^2^ ≤ 0.010).

**FIGURE 7:**
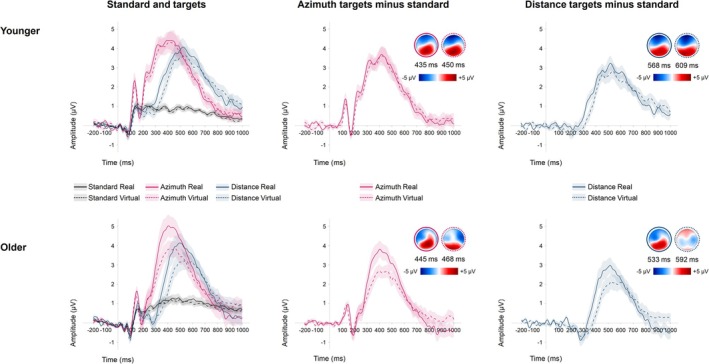
Grand‐averaged ERP waveforms recorded in the real (solid lines) and virtual (dashed lines) environments for both age groups at electrodes CP1, CPz, CP2, P1, Pz, and P2. Left panel: ERPs elicited by standard and target stimuli. Middle and right panels: ERP difference waveforms (target minus standard) showing the P3b. Shaded areas beyond the waveforms refer to the range of ± one standard error. Topographic maps refer to the point in time when the area of the respective component reaches 50% (FAL).

Figure 7 compares the P3b between environments, separated by target dimension and age group, including topographic maps at the timepoint of the FAL.

#### 3.2.5 Additional Analyses

##### 3.2.5.1 Null Hypotheses Testing

To test the hypothesis that target sounds elicit comparable neurophysiological responses in real and virtual environments, we additionally performed a null hypothesis significance testing with BF analyses (see Section 2.6), comparing ERP amplitudes and latencies across environments for each target dimension and age group (see Table 1).

**TABLE 1:** Pairwise comparisons of the ERPs' amplitudes and FAL between the real and virtual environments for the younger and the older age groups using Bayesian factors for null hypothesis testing. Moderate evidences in the direction of H_0_ (1/10 < BF < 1/3) are shown in bold.

	P1	N1	P2	MMN		P3b	
	Peak amplitude	Peak amplitude	Peak amplitude	Mean amplitude	FAL	Mean amplitude	FAL
**Real** azimuth younger vs. **virtual** azimuth younger	**0.16**	**0.22**	**0.14**	1.53	**0.28**	**0.14**	**0.21**
**Real** distance younger vs. **virtual** distance younger	**0.21**	0.43	**0.17**	4.07	**0.25**	0.41	1.54
**Real** azimuth older vs. **virtual** azimuth older	**0.16**	**0.21**	4.50	0.51	1.41	38.85	**0.32**
**Real** distance older vs. **virtual** distance older	**0.19**	**0.24**	**0.14**	**0.24**	0.71	34.19	50.31

Consistent with the ANOVAs, the BF analyses for P1 showed moderate evidence against the alternative hypothesis (H_1_), suggesting comparable early sensory processing of sound stimuli in both environments. For N1, the BFs also indicated slight to moderate evidence against H_1_, aligning with the findings for P1. Although the ANOVA showed a marginally nonsignificant main effect of environment on P2 amplitudes (*F*_1,42_ = 3.79, *p* = 0.058, *ƞ*_*p*_^2^ = 0.083), BF provided moderate evidence supporting a difference in amplitude between the real and virtual environments for azimuth targets in older participants. Regarding MMN, the BFs showed slight to moderate evidence in favor of H_1_ for younger participants, suggesting higher amplitudes in the real compared with the virtual environment. However, the latencies of the MMN were comparable, with moderate evidence for H_0_ among younger participants. For older participants, there was slight evidence in the direction of H_1_ when comparing latencies for azimuth targets. For P3b, the BFs indicated strong evidence for differences in mean amplitude between real and virtual environments in older participants for both azimuth and distance targets. Strong evidence for differences in P3b latency was also found for distance targets among older participants. A moderate BF was observed for the mean amplitude of distance targets among younger participants, suggesting weak evidence for a difference. All other comparisons showed evidence favoring H_0_.

In summary, most pairwise comparisons showed BFs indicating comparable neurophysiological responses between real and virtual environments. Exceptions included P2 amplitude for azimuth targets in older participants, MMN amplitude among younger participants, and P3b amplitude and latencies in older participants.

##### 3.2.5.2 Brain–Behavior Correlations

As an additional, explorative analysis of potential relationships between behavioral and brain measures, we calculated correlations between accuracy in change detection and the mean amplitudes and FAL of the MMN and P3b, as well as between the MMN and P3b measures, separately for each age group. Except for a few moderate effects, no clear patterns of relationships were observed between the behavioral and neural measures, or between the amplitudes and latencies of the MMN and P3b. The results are provided in the Supporting Information S1 and S2.

##### 3.2.5.3 Analyses With Unaveraged Near and Far Distance Dimension

To additionally gain insights into the perception and processing of distance changes, we conducted all analyses by considering the near and far sound positions separately, without averaging the responses from the two positions. Overall, however, no substantial differences were found compared with the analyses with the averaged distance dimension. This result makes it less likely that the distance effect primarily results from an increase in loudness (position change to the near loudspeaker) but rather results from a (loudness) cue reflecting a change in distance with respect to the reference. The results are provided in the Supporting Information S3–S11.

## 4 Discussion

The main goal of this study was to verify if an audiovisual virtual test environment can reliably replicate how people perceive and process changes in sound position, both in azimuth and distance, and how this is affected by age. Therefore, younger and older participants performed an auditory change detection task in two settings: a real environment, where sounds were played through physical loudspeakers, and a virtual environment, where auditory stimuli were played back via headphones and a visual replica of the laboratory room was presented via a HMD. In both environments, participants had to respond to any changes in sound position, either in distance or azimuth, relative to a central reference point, and both behavioral (detection accuracy) and electrophysiological data (ERPs) were measured. If comparable results are observed between the real and virtual environments, this would provide strong evidence supporting the validity and reliability of the virtual setup for studying both general auditory spatial processing and age‐related differences.

### 4.1 Accuracy of Target Detection in Both Environments

Participants demonstrated significantly higher accuracy in detecting position changes of sounds in azimuth (left and right) compared with changes in distance (near and far). These findings underscore the robustness of sound localization in azimuth, supporting previous research suggesting that humans exhibit greater sensitivity to directional cues than to distance cues such as the DRR or the loudness (Arend et al. 2021; Kolarik et al. 2016). Importantly, despite the expected age‐related sensory declines, older participants demonstrated comparable detection accuracy to younger participants. Neither age significantly influenced detection accuracy, nor any interaction effect was observed. Thus, we found no statistical evidence that age‐related sensory declines affected older participants' ability to detect auditory position changes, despite their higher hearing loss, particularly at the higher frequencies (see Figure 1). Although high frequencies (above 1 kHz) are important for an accurate sound localization and age‐related hearing loss can influence spatial perception (Glyde et al. 2013; Russell 2022), these factors did not significantly impact performance in our study. Since the task focused on detecting changes in sound position relative to a reference, rather than judging absolute distances or directional angles, the higher hearing thresholds in older participants may have had a minimal effect. Moreover, the comparable performance of participants in both the real and virtual environments indicates that the virtual setup reliably replicated the real‐world laboratory conditions.

### 4.2 ERPs Related to Detection of Target Sound Locations

The topographies of the P1‐N1‐P2 complex displayed a clear frontocentral distribution for both standard and target stimuli across age groups and environments, consistent with previous findings on auditory evoked potentials (Näätänen and Picton 1987). The similarity in topographical patterns between the real and virtual environments suggests that the virtual environment successfully replicated the neural processes involved in early auditory perception. The P1 response showed consistent amplitude when comparing between environments and target dimensions, emphasizing its primary role in encoding the sensory characteristics of stimuli (Eggermont and Ponton 2002; Grunwald et al. 2003).

The N1 amplitude was significantly higher in older participants, especially within the virtual environment, potentially reflecting an age‐related compensatory response as older adults often require enhanced attentional mechanisms for auditory processing (Alain and Woods 1999; Amenedo and Díaz 1998; Finnigan et al. 2011; Strömmer et al. 2017). This seems to be particularly pronounced under difficult listening conditions, for example, in the comprehension of degraded speech, where an increased N1 with age could indicate a higher ‘neural effort’ via resource allocation (e.g., Kuruvilla‐Mathew et al. 2022; Obleser and Kotz 2011).

Similarly, for the P2 component, a pronounced age effect was observed across both environments, with older participants showing larger amplitudes than younger ones (Bertoli et al. 2002). As for the N1, this increase may reflect compensatory cognitive mechanisms, as older adults tend to engage in more extensive auditory processing to offset sensory declines (Amenedo and Díaz 1998; Arnott et al. 2011; Cabeza et al. 2002; Strömmer et al. 2017). This pattern suggests that older adults allocate additional cognitive resources to process spatial sound changes.

Overall, these findings indicate that our virtual setup does not compromise the robustness of these early auditory ERP markers, supporting its feasibility for studying sensory processes in auditory spatial perception. The consistent age effects observed across the real and virtual environments also underscore the virtual environments' reliability in capturing age‐dependent differences in auditory processing. Specifically, older adults demonstrated higher N1 and P2 amplitudes across environments, suggesting compensatory mechanisms involving enhanced attention or additional cognitive resources for auditory processing (e.g., Alain and Woods 1999; Amenedo and Díaz 1998; Bertoli et al. 2002; Pichora‐Fuller 2008). The additional null hypothesis significance testing showed that most pairwise comparisons yielded BFs indicating comparable neurophysiological responses between real and virtual environments. However, for the P2, BFs provided moderate evidence in favor of H_1_ for azimuth targets among older adults, suggesting slight differences that may reflect subtle perceptual changes or attentional modulation (Picton and Hillyard 1974), with the virtual environment potentially altering auditory processing. Notably, no amplitude differences were found for the two target dimensions, suggesting that the underlying early neural mechanisms are similarly engaged by spatial changes in both azimuth and distance, irrespective of age or environment.

Across both environments, auditory spatial changes were reliably indicated by the MMN, consistent with its role as a robust marker of deviance detection (Chung and Park 2018). Topographies showed a predominantly frontal distribution of the MMN with a slight right‐hemispheric bias. This frontal distribution aligns with the view that the MMN reflects preattentive change detection processes originating in the auditory cortex and frontal regions (Näätänen et al. 2007). The right‐hemispheric bias observed in our study is consistent with previous research suggesting right‐hemispheric dominance in spatial auditory processing (Jiao et al. 2021; Palomäki et al. 2005). Importantly, the MMN topographies were largely similar between the real and virtual environments, also indicating that the virtual setup effectively captured the neural mechanisms underlying automatic auditory change detection.

The MMN amplitude was increased for changes in azimuth compared with distance. Since MMN amplitudes are known to increase with greater discriminability between the deviant and the standard sound (Fitzgerald and Todd 2020), this finding aligns with our behavioral results, which showed better detection of azimuth targets compared with distance targets. This observation is also consistent with previous research suggesting that listeners have greater sensitivity to binaural than to monaural cues (Arend et al. 2021; Kolarik et al. 2016). Additionally, we found that the MMN amplitude was reduced in the virtual environment compared with the real environment. The BF analyses showed that this was mostly the case among younger adults. Although participants showed similar detection accuracy in both environments, the auditory changes in the virtual setting may have been less salient or less accurately represented compared with the real loudspeaker setup. This reduced salience might have made the auditory changes harder to detect, leading to a reduced MMN amplitude (Fiedler et al. 2011; Winkler et al. 1992). Another potential explanation is that the virtual environment imposed a higher cognitive load on participants, as they were wearing an HMD, an experience that is not typically part of their everyday activities. An increased cognitive load, especially when induced by high visual task demands, has been shown to diminish MMN amplitudes (Haroush et al. 2010). Therefore, the novelty and additional demands of using an HMD may have contributed to the observed reduction in MMN amplitude in the virtual environment.

Furthermore, when comparing age groups, we found that the MMN amplitude was more pronounced in younger participants than in older ones. This age‐related difference is consistent with previous findings that MMN amplitudes tend to decrease with aging, likely due to age‐related declines in auditory processing and neural plasticity (Bartha‐Doering et al. 2015; Cheng et al. 2013; Ruzzoli et al. 2012; Getzmann et al. 2024). Notably, the reduced MMN observed in the virtual environment and for distance targets was consistent across both age groups, with no significant interaction effects. This indicates that aging neither enhanced the reduction in MMN amplitude within the virtual environment nor influenced the distinction between azimuth and distance targets.

Moreover, the MMN occurred later for distance targets compared with azimuth targets, and it was also delayed in the virtual environment relative to the real environment. Similar to its amplitude, MMN latency is shorter when the standard and target sounds are more distinct and longer when the stimuli are less discriminable (Näätänen et al. 2007). A lower discriminability likely increases the time point at which an uncommon change is detected (Fitzgerald and Todd 2020). These findings suggest that changes in distance were less salient than changes in azimuth, consistent with both the behavioral and amplitude data. Furthermore, changes in the virtual environment were less salient than those in the real environment, as also reflected in the MMN amplitude. Despite these findings, no behavioral differences in auditory change detection were observed between environments. This suggests that although the MMN showed a delay and reduced salience for changes in VR (especially for distance changes), these effects did not significantly affect behavior, potentially due to increased attention compensating for reduced salience. BF analyses further revealed no clear evidence for differences in MMN latency between the environments, supporting the lack of behavioral changes.

The P3b component exhibited the expected parietal distribution, with slight variations depending on the target dimension. This parietal focus is consistent with the understanding of the P3b as reflecting attentional allocation and stimulus evaluation processes (Polich 2007; Wronka et al. 2012). The similarity in P3b topographies between real and virtual environments further supports the virtual setup's validity in capturing higher‐order cognitive processes linked to auditory spatial attention. Notably, in older participants across both environments, we observed a shift in P3b topography toward more anterior brain regions. This shift likely reflects an increased reliance on executive control processes for categorization and updating, serving as a compensatory mechanism to maintain cognitive task performance (Fjell and Walhovd 2004; O'Connell et al. 2012), as also proposed in the context of the PASA hypothesis (Posterior–Anterior Shift in Aging; Davis et al. 2008; for review, Grady 2012).

Regarding the amplitude of the P3b, a significant main effect of target dimension was observed, with amplitudes more pronounced for azimuth than distance targets. This finding suggests that lateral stimuli capture attention more effectively than distance stimuli, likely due to the greater ecological relevance of directional information in everyday auditory scenes (de la Torre‐Ortiz et al. 2023). Additionally, humans generally exhibit better perceptual resolution for azimuth than for distance in auditory localization, potentially leading to stronger neural responses (Kolarik et al. 2016). Furthermore, the integration of binaural cues, such as interaural time and level differences, plays a crucial role in azimuth perception. In contrast, the perception of egocentric distance changes relies mostly on cues that are independent of interaural changes, such as intensity and timbre variations (Zahorik et al. 2005). The enhanced processing of binaural, compared with monaural, information may contribute to more robust neural responses, resulting in larger P3b amplitudes. This pattern of results aligns with previous research on other auditory ERP components, such as the MMN, which has demonstrated greater sensitivity to changes in sound location compared with other auditory features (Tata and Ward 2005; Y. Wang et al. 2022).

Further, we observed higher P3b amplitudes in the real environment compared with the virtual environment, supporting prior assumptions about a diminished P3b when the task is more challenging (Katayama and Polich 1998; Verleger et al. 2014). However, this effect appears to be driven primarily by older participants, who showed descriptively lower P3b amplitudes in the virtual compared with the real environment for both azimuth and distance targets. In contrast, younger participants showed no differences in P3b amplitude between the environments. This observation is further supported by marginally significant interaction effects between age and environment, as well as between age, environment, and target dimension, along with BF analyses. The results suggest that the difference in P3b amplitude between environments was more pronounced in older participants, who showed a larger mean difference between the real and virtual environments, with the latter showing a reduced amplitude. This implies that the virtual environment may demand greater cognitive resources, particularly for older adults. Although our analysis revealed no significant age‐related reduction in P3b responses overall, the aforementioned tendencies and BF analyses support previous research where older adults showed a diminished deviance‐related P3b amplitude, often associated with age‐related declines in stimulus processing (Getzmann et al. 2024; Porcaro et al. 2019). Several factors may explain why this is particularly pronounced in the virtual environment. For instance, the real environment likely provides more familiar, ecologically valid cues, which could reduce cognitive load, especially for older adults. Furthermore, real‐world settings offer richer multisensory information, which might benefit older adults who often rely more on multisensory integration due to declines in individual sensory modalities (Hirst et al. 2019). In line with that, the Compensation‐Related Utilization of Neural Circuits Hypothesis (CRUNCH) posits that older individuals recruit additional neural resources to achieve similar performance levels as younger adults (Reuter‐Lorenz and Cappell 2008; Reuter‐Lorenz and Lustig 2005). It may be possible that older adults are more capable of employing such compensatory mechanisms to maintain performance in more familiar environments. Additionally, older adults may have less familiarity with VR technology, which could increase cognitive load when interpreting the virtual environment (Roberts et al. 2019).

The P3b latency was longer for distance compared with azimuth targets and was also delayed in the virtual compared with the real environment. This suggests that processing auditory depth cues, especially in the virtual setting, requires increased cognitive effort. This is further emphasized by the observed interaction effect between the environment and target dimension. Here, BF analyses revealed that for distance targets, the P3b was more delayed in the virtual compared with the real environment within both age groups. This delay may be due to the absence of natural spatial cues in the virtual environment, which could necessitate additional cognitive processing to integrate auditory information (Stodt et al. 2024).

In conclusion, the results demonstrate a high degree of comparability between the real and virtual environments in terms of auditory processing, with similar topographical patterns and age‐related effects observed across both setups. Notably, the virtual environment was effective in replicating early auditory processing, including components such as the P1, N1, and P2. Further, no significant effects of target dimension were found, indicating that the observed age‐related differences in early auditory processing were consistent across both azimuth and distance targets. This suggests that compensatory mechanisms engaged by older adults may apply broadly, regardless of the specific spatial dimension.

Nonetheless, some distinct characteristics emerged, particularly in the MMN and P3b components, where a reduction in amplitude and latency delay were observed in the virtual environment. These differences may reflect the reduced salience of auditory cues or increased cognitive load associated with the virtual setting, especially for older participants. Importantly, age‐related effects were evident across environments, with older adults showing reduced MMN and P3b amplitudes, indicating declines in sensory processing and attentional allocation with age. Additionally, older adults showed a tendency for lower P3b amplitudes in the virtual environment compared with the real environment, suggesting that the virtual setup may impose greater cognitive demands on this age group. However, despite these differences, the virtual environment still reliably captured the neural mechanisms underlying auditory spatial perception, making it a valid and reliable tool for studying auditory processing in both younger and older adults. Notably, differences were also found between target dimensions, with a reduced and delayed MMN as well as a reduced P3b for distance changes, suggesting that distance changes may require more cognitive resources, as they are less salient and more challenging to process.

### 4.3 Limitations and Outlook

Several limitations of the present study should be acknowledged. First, although the use of generic BRIRs in virtual environments has been shown to adequately represent auditory spatial cues, particularly in static setups (Berger et al. 2018; Blau et al. 2021; Zahorik 2000), the detection and processing of changes in sound positions may be improved with individualized BRIRs. These offer more precise spatial localization cues, potentially enhancing the accuracy of sound position detection (Mehra et al. 2016). Future studies could investigate detection capabilities by incorporating individualized BRIRs or adaptive algorithms that tailor spatial cues to each participant's unique auditory profile.

Additionally, the visual replication of the laboratory environment in a virtual environment could have had an impact on the audiovisual perception of the scene. In real‐world settings, visual depth information plays a crucial role in enhancing auditory localization through multimodal integration. However, in VR, this effect may be diminished if visual and auditory cues are not accurately aligned (Paquier et al. 2016). Although the present study did not specifically examine the influence of the room's visual presentation, it is reasonable to assume that the visual reference may have contributed to distance perception, albeit in a relative sense. Prior research suggests that people tend to underestimate room size in visual‐only virtual environments but overestimate it in auditory‐only environments, with the most accurate spatial perception occurring when both auditory and visual cues are present (Larsson et al. 2007). Future VR research could therefore focus on enhancing visual realism to better support this auditory–visual integration. Additionally, incorporating head‐tracking technology could enable the dynamic adjustment of spatial cues in response to head movements, offering a more immersive auditory experience consistent with real‐world presence and auditory processing (Begault et al. 2001).

Another consideration is the potential cognitive load associated with VR systems. Participants, particularly older adults, may experience cognitive fatigue or visual strain due to prolonged use of head‐mounted displays, which could affect performance (Brown et al. 2022). Further research could explore the impact of these factors on participant engagement and accuracy.

Further, although this study controlled for hearing loss, it is important to recognize that the findings may not fully represent the experiences of individuals with a wider range of hearing capabilities. Future research could include more diverse age groups and individuals with hearing impairments to improve the generalizability of the results.

In addition, overall accuracy levels were relatively high. Although we applied a logit transformation to mitigate distortions caused by extreme values, the high performance level may still have limited the detection of subtle differences.

Lastly, the use of pink noise in this study represents an artificial sound not commonly encountered in real‐world environments. Future studies should investigate localization and attention mechanisms in more ecologically valid contexts, such as multi‐talker environments using speech signals.

## 5 Conclusion

This study validated the use of an audiovisual virtual test environment to study auditory spatial attention and sound localization in both younger and older adults, by comparing behavioral and neurophysiological results with those from a real‐world setup. Results showed that participants accurately detected changes in sound position, but changes in distance were less often correctly detected than changes in azimuth. Similar neural responses were found across real and virtual environments, although some differences emerged, notably in the MMN and P3b responses, which were less pronounced in the virtual setting and for distance targets. These discrepancies may be due to the increased cognitive load or the reduced salience of auditory cues. Furthermore, older adults tended to show lower P3b amplitudes in the virtual environment compared with the real environment, suggesting that the virtual setup may impose greater cognitive demands on this age group.

Future applications of such a virtual test environment could enhance our understanding of how individuals process auditory cues in real‐world tasks. Simulating complex environments could help investigate age‐related neural mechanisms and attentional processes in dynamic situations. This would offer insights into how older individuals can be supported in challenging tasks, such as identifying sounds in noisy environments. Enhancements like individualized spatial cues and head‐tracking could improve immersion, helping individuals adapt to various auditory conditions while supporting cognitive training and safety solutions.

## Author Contributions

**Benjamin Stodt:** conceptualization, data curation, formal analysis, investigation, methodology, project administration, software, validation, visualization, writing – original draft, writing – review and editing. **Daniel Neudek:** conceptualization, methodology, software, writing – review and editing. **Rainer Martin:** conceptualization, funding acquisition, methodology, resources, supervision, writing – review and editing. **Edmund Wascher:** resources, writing – review and editing. **Stephan Getzmann:** conceptualization, formal analysis, funding acquisition, methodology, supervision, writing – review and editing.

## Conflicts of Interest

The authors declare no conflicts of interest.

### Peer Review

The peer review history for this article is available at https://www.webofscience.com/api/gateway/wos/peer‐review/10.1111/ejn.70141.

## Supporting information


**Table S1.** Spearman correlations between accuracy in change detections and the mean amplitudes and fractional area latencies (FAL) of the MMN and P3b, for each environment and target dimension, separated by age group (* *p* ≤ 0.050). Significance values were corrected based on the jackknifing procedure.
**Table S2.** Spearman correlations between the mean amplitudes and fractional area latencies (FAL) of the MMN and P3b, for each environment and target dimension, separated by age group (** *p* ≤ 0.010). Significance values were corrected based on the jackknifing procedure.
**Table S3.** Results of the ANOVA for the effects of age (younger, older), environment (real, virtual), and target dimension (azimuth, near, far), and their interactions on detection accuracy, P1, N1, and P2 peak amplitudes as well as MMN and P3b mean amplitudes and fractional area latencies (FAL). *F*‐ and *p*‐values were corrected based on the jackknifing procedure. Significant effects are marked in bold.
**Figure S4.** Accuracy in detecting azimuth, near, and far targets, shown separately for both environments and age groups. Horizontal bold lines represent the mean values of performance over all participants, vertical bars represent ± one standard deviation, and dots indicate the individual mean accuracy values.
**Table S5.** Pairwise post hoc analysis of the effects of target dimension (azimuth, near, far) on detection accuracy. Accuracy was logit‐transformed prior to analyses. *p*‐values were corrected for multiple testing using false discovery rate correction.
**Figure S6.** Grand averaged ERP waveforms from standard (center position) and target (azimuth, near, far position) trials at electrodes Fz, F1, F2, FCz, FC1, and FC2, comparing the P1‐N1‐P2 complex in the real (solid lines) and virtual environment (dashed lines) for both age groups. Shaded areas beyond the waveforms refer to the range of ± one standard error.
**Table S7.** Pairwise post hoc analysis of the interaction effect of age (younger, older) and environment (real, virtual) on N1 peak amplitude, comparing mean differences. *T*‐ and *p*‐values were corrected based on the jackknifing procedure.
**Table S8.** Pairwise post hoc analysis of the interaction effect of environment (real, virtual) and target dimension (azimuth, near, far) on N1 peak amplitude, comparing mean differences. *T*‐ and *p*‐values were corrected based on the jackknifing procedure. *p*‐values were corrected for multiple testing using false discovery rate correction.
**Figure S9.** Grand‐averaged ERP waveforms recorded in the real (solid lines) and virtual (dashed lines) environments for both age groups at electrodes AF4, AF8, Fz, F2, F4, F6, FCz, FC2, FC4, FT8, Cz, C2, C4, C6, and T8. First panel: ERPs elicited by standard and target stimuli. Second to fourth panels: ERP difference waveforms (target minus standard) showing the MMN evoked by sound changes to azimuth, near, and far targets. Shaded areas beyond the waveforms refer to the range of ± one standard error. Topographic maps refer to the point in time when the area of the respective component reaches 50% (FAL).
**Figure S10.** Grand‐averaged ERP waveforms recorded in the real (solid lines) and virtual (dashed lines) environments for both age groups at electrodes CP1, CPz, CP2, P1, Pz, and P2. Left panel: ERPs elicited by standard and target stimuli. First panel: ERPs elicited by standard and target stimuli. Second to fourth panels: ERP difference waveforms (target minus standard) showing the P3b evoked by sound changes to azimuth, near, and far targets. Shaded areas beyond the waveforms refer to the range of ± one standard error. Topographic maps refer to the point in time when the area of the respective component reaches 50% (FAL).
**Table S11.** Pairwise post hoc analysis of the effect of target dimension (azimuth, near, far) on MMN and P3b mean amplitudes and fractional area latencies (FAL). *T*‐ and *p*‐values were corrected based on the jackknifing procedure. *p*‐values were corrected for multiple testing using false discovery rate correction.

## Data Availability

Data will be made available on request.
