# Supplementary material for: Age‐Related Differences in Neural Correlates of Auditory Spatial Change Detection in Real and Virtual Environments
Source: Eur J Neurosci. 2025 May 15;61(10):e70141. doi: 10.1111/ejn.70141 (PMC12081944; doi:10.1111/ejn.70141)
Supplement: Supplementary file 1 — Table S1. Spearman correlations between accuracy in change detections and the mean amplitudes and fractional area latencies (FAL) of the MMN and P3b, for each environment and target dimension, separated by age group (* p ≤ 0.050). Significance values were corrected based on the jackknifing procedure. Table S2. Spearman correlations between the mean amplitudes and fractional area latencies (FAL) of the MMN and P3b, for each environment and target dimension, separated by age group (** p ≤ 0.010). Significance values were corrected based on the jackknifing procedure. Table S3. Results of the ANOVA for the effects of age (younger, older), environment (real, virtual), and target dimension (azimuth, near, far), and their interactions on detection accuracy, P1, N1, and P2 peak amplitudes as well as MMN and P3b mean amplitudes and fractional area latencies (FAL). F‐ and p‐values were corrected based on the jackknifing procedure. Significant effects are marked in bold. Figure S4. Accuracy in detecting azimuth, near, and far targets, shown separately for both environments and age groups. Horizontal bold lines represent the mean values of performance over all participants, vertical bars represent ± one standard deviation, and dots indicate the individual mean accuracy values. Table S5. Pairwise post hoc analysis of the effects of target dimension (azimuth, near, far) on detection accuracy. Accuracy was logit‐transformed prior to analyses. p‐values were corrected for multiple testing using false discovery rate correction. Figure S6. Grand averaged ERP waveforms from standard (center position) and target (azimuth, near, far position) trials at electrodes Fz, F1, F2, FCz, FC1, and FC2, comparing the P1‐N1‐P2 complex in the real (solid lines) and virtual environment (dashed lines) for both age groups. Shaded areas beyond the waveforms refer to the range of ± one standard error. Table S7. Pairwise post hoc analysis of the interaction effect of age (younger, older) and environment ( [file EJN-61-0-s001.pdf]

**Supplements**

Table S1. Spearman correlations between accuracy in change detections and the mean amplitudes and fractional area latencies (FAL) of the MMN and P3b, for each environment and target dimension, separated by age group (* $p \leq .050$). Significance values were corrected based on the jackknifing procedure.

| | **MMN** | | | | **P3b** | | | |
|---|---|---|---|---|---|---|---|---|
| | **Mean Amplitude** | | **FAL** | | **Mean Amplitude** | | **FAL** | |
| Accuracy / Target Dimension | Young | Old | Young | Old | Young | Old | Young | Old |
| Real Azimuth | **-.467 *** | -.212 | .199 | .077 | **-.465 *** | -.095 | .165 | .180 |
| Real Distance | -.145 | **-.417 *** | .288 | -.229 | -.069 | -.068 | .261 | .237 |
| Virtual Azimuth | -.073 | -.381 | .289 | .228 | **.496 *** | .196 | .151 | .319 |
| Virtual Distance | -.138 | -.196 | .201 | .200 | .363 | .295 | -.026 | .370 |

Table S2. Spearman correlations between the mean amplitudes and fractional area latencies (FAL) of the MMN and P3b, for each environment and target dimension, separated by age group (** $p \leq .010$). Significance values were corrected based on the jackknifing procedure.

| | | **P3b** | |
|---|---|---|---|
| | | **Mean Amplitude** | |
| **MMN** | Target Dimension | Young | Old |
| **Mean Amplitude** | Real Azimuth | **.612 **** | .002 |
| | Real Distance | .176 | -.397 |
| | Virtual Azimuth | .416 | -.072 |
| | Virtual Distance | .298 | -.120 |
| | | **FAL** | |
| **FAL** | Real Azimuth | .297 | -.110 |
| | Real Distance | -.131 | -.131 |
| | Virtual Azimuth | -.321 | .083 |
| | Virtual Distance | .144 | -.004 |

Table S3. Results of the ANOVA for the effects of age (younger, older), environment (real, virtual), and target dimension (azimuth, near, far), and their interactions on detection accuracy, P1, N1, and P2 peak amplitudes as well as MMN and P3b mean amplitudes and fractional area latencies (FAL). F- and p-values were corrected based on the jackknifing procedure. Significant effects are marked in bold.

| | | | | **P1** | | | **N1** | | | **P2** | | | **MMN** | | | | | | **P3b** | | | | | |
|---|---|---|---|---|---|---|---|---|---|---|---|---|---|---|---|---|---|---|---|---|---|---|---|---|
| | **Accuracy** | | | **Peak Amplitude** | | | **Peak Amplitude** | | | **Peak Amplitude** | | | **Mean Amplitude** | | | **FAL** | | | **Mean Amplitude** | | | **FAL** | | |
| **Effect** | *F* | *p* | $\eta_p^2$ | *F* | *p* | $\eta_p^2$ | *F* | *p* | $\eta_p^2$ | *F* | *p* | $\eta_p^2$ | *F* | *p* | $\eta_p^2$ | *F* | *p* | $\eta_p^2$ | *F* | *p* | $\eta_p^2$ | *F* | *p* | $\eta_p^2$ |
| Age | 1.24 | .272 | .029 | 3.48 | .069 | .076 | **5.43** | **.025** | **.114** | **9.21** | **.004** | **.180** | **4.46** | **.041** | **.096** | 0.41 | .528 | .010 | 0.51 | .481 | .012 | 0.10 | .757 | .002 |
| Environment | 3.37 | .073 | .074 | 1.95 | .170 | .044 | 0.02 | .896 | <.001 | 2.93 | .094 | .065 | **8.63** | **.005** | **.170** | 1.65 | .206 | .038 | **1.93** | **.002** | **.206** | **16.49** | **<.001** | **.282** |
| Target Dimension | **17.13** | **<.001** | **.290** | 0.59 | .558 | .014 | 1.01 | .370 | .023 | 1.54 | .221 | .035 | **31.32** | **<.001** | **.427** | **3.93** | **.023** | **.085** | **8.94** | **<.001** | **.176** | **161.82** | **<.001** | **.794** |
| Age X Environment | 1.05 | .312 | .024 | <0.01 | .977 | <.001 | **4.70** | **.036** | **.101** | 0.40 | .531 | .009 | 1.27 | .267 | .029 | 0.01 | .905 | <.001 | 1.13 | .294 | .026 | 0.48 | .491 | .011 |
| Age X Target Dim. | 0.25 | .780 | .006 | 0.81 | .450 | .019 | 0.07 | .929 | .002 | 1.25 | .293 | .029 | 0.25 | .779 | .006 | 0.52 | .595 | .012 | 0.58 | .562 | .014 | 2.23 | .114 | .050 |
| Environment X Target Dim. | 2.69 | .074 | .060 | 0.46 | .631 | .011 | **4.41** | **.015** | **.095** | 0.54 | .587 | .013 | 1.42 | .247 | .033 | 0.93 | .400 | .022 | 0.33 | .719 | .008 | 2.12 | .126 | .048 |
| Age X Environment X Target Dim. | 0.14 | .867 | .003 | 1.43 | .246 | .033 | 0.05 | .953 | .001 | 0.94 | .395 | .022 | 2.86 | .063 | .064 | 0.04 | .960 | .001 | 2.64 | .077 | .059 | 0.31 | .735 | .007 |

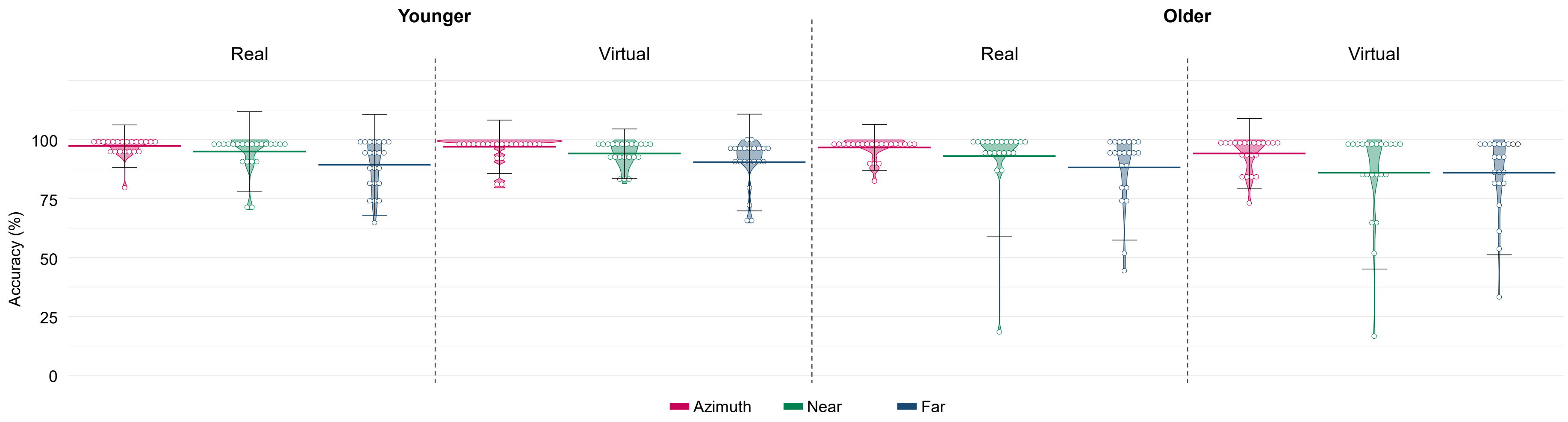


Figure S4. Accuracy in detecting azimuth, near, and far targets, shown separately for both environments and age groups. Horizontal bold lines represent the mean values of performance over all participants, vertical bars represent ± one standard deviation, and dots indicate the individual mean accuracy values.

Table S5. Pairwise post-hoc analysis of the effects of target dimension (azimuth, near, far) on detection accuracy. Accuracy was logit-transformed prior to analyses. P-values were corrected for multiple testing using False Discovery Rate correction.

| | $M_1$ ($SD_1$) | $M_2$ ($SD_2$) | *T* | *p* |
|---|---|---|---|---|
| **Azimuth vs. Near** | 96.28 (5.77) | 92.05 (14.41) | 3.43 | .002 |
| **Azimuth vs. Far** | 96.28 (5.77) | 88.49 (13.61) | 6.82 | <.001 |
| **Near vs. Far** | 92.05 (14.41) | 88.49 (13.61) | 2.42 | .020 |

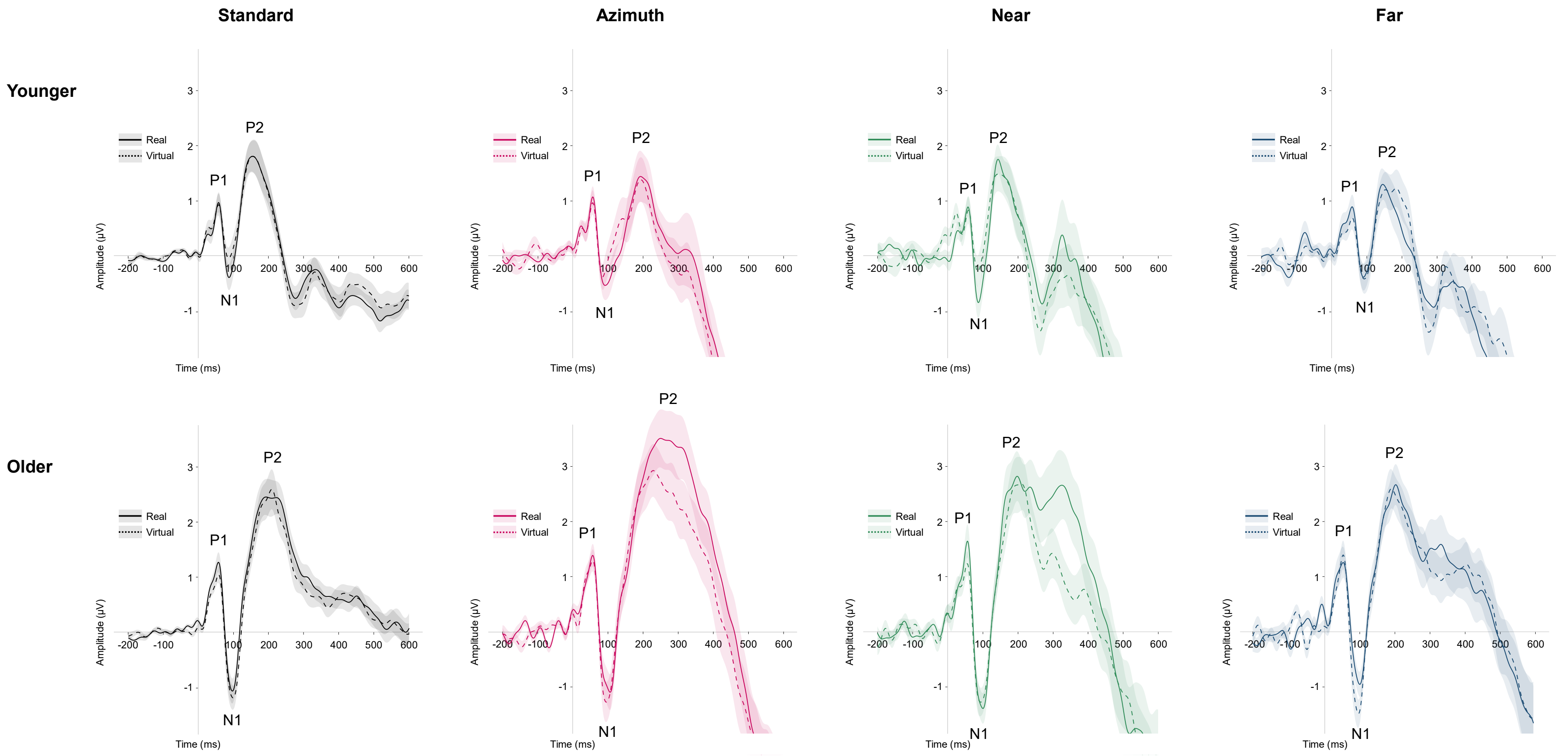


Figure S6. Grand averaged ERP waveforms from standard (center position) and target (azimuth, near, far position) trials at electrodes Fz, F1, F2, FCz, FC1, and FC2, comparing the P1-N1-P2 complex in the real (solid lines) and virtual environment (dashed lines) for both age groups. Shaded areas beyond the waveforms refer to the range of +/- one standard error.

Table S7. Pairwise post-hoc analysis of the interaction effect of age (younger, older) and environment (real, virtual) on N1 peak amplitude, comparing mean differences. T- and p-values were corrected based on the jackknifing procedure.

| | $M_{diff, real}$ ($SD_{diff,real}$) | $M_{diff,virtual}$ ($SD_{diff,virtual}$) | *T* | *p* |
|---|---|---|---|---|
| **Real vs. Virtual** | 0.23 (0.03) | -0.20 (0.03) | 2.17 | .036 |

Table S8. Pairwise post-hoc analysis of the interaction effect of environment (real, virtual) and target dimension (azimuth, near, far) on N1 peak amplitude, comparing mean differences. T- and p-values were corrected based on the jackknifing procedure. P-values were corrected for multiple testing using False Discovery Rate correction.

| | $M_{diff1}$ ($SD_{diff1}$) | $M_{diff2}$ ($SD_{diff2}$) | *T* | *p* |
|---|---|---|---|---|
| **Azimuth vs. Near** | -0.01 (0.19) | 0.33 (0.23) | -0.62 | .599 |
| **Azimuth vs. Far** | -0.01 (0.19) | -0.28 (0.25) | 0.53 | .599 |
| **Near vs. Far** | 0.33 (0.23) | -0.28 (0.25) | 1.30 | .597 |

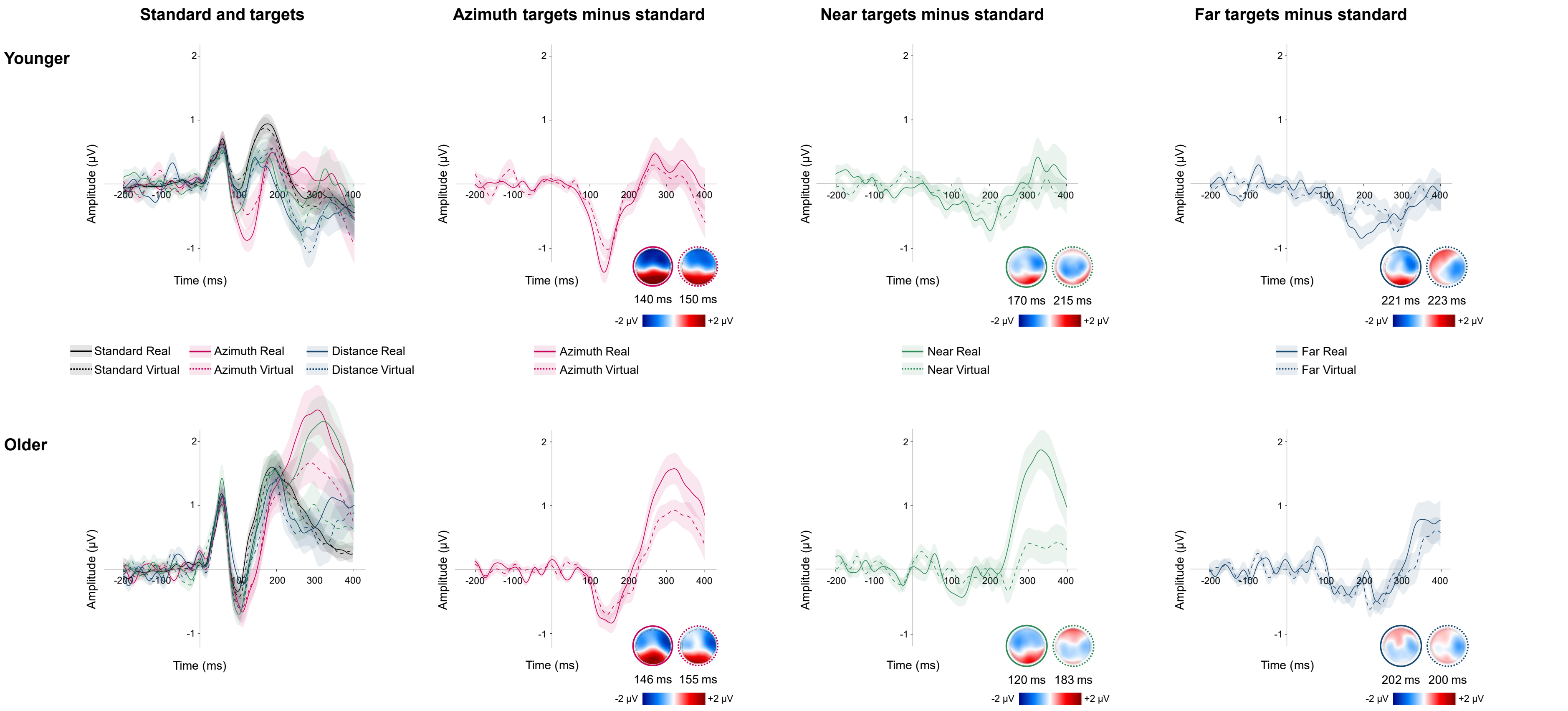


Figure S9. Grand-averaged ERP waveforms recorded in the real (solid lines) and virtual (dashed lines) environments for both age groups at electrodes AF4, AF8, Fz, F2, F4, F6, FCz, FC2, FC4, FT8, Cz, C2, C4, C6, and T8. First panel: ERPs elicited by standard and target stimuli. Second to fourth panels: ERP difference waveforms (target minus standard) showing the MMN evoked by sound changes to azimuth, near, and far targets. Shaded areas beyond the waveforms refer to the range of +/- one standard error. Topographic maps refer to the point in time when the area of the respective component reaches 50 percent (FAL).

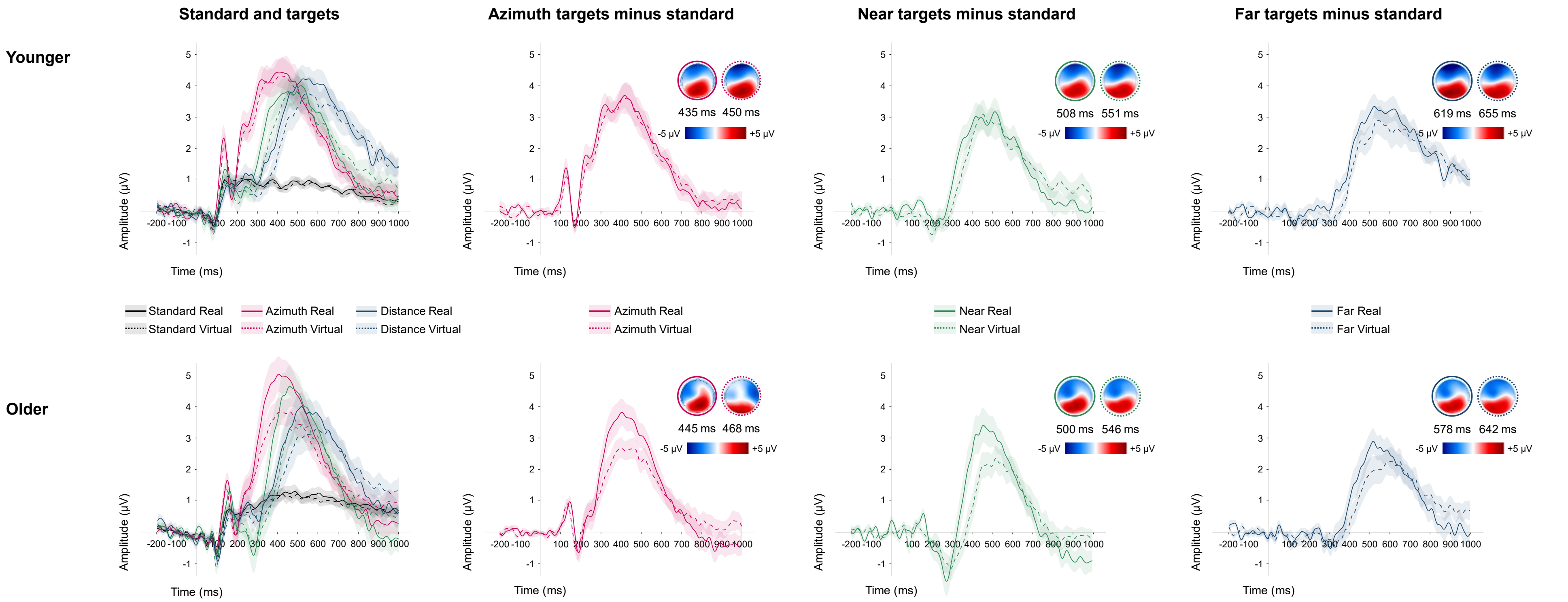


Figure S10. Grand-averaged ERP waveforms recorded in the real (solid lines) and virtual (dashed lines) environments for both age groups at electrodes CP1, CPz, CP2, P1, Pz, and P2 Left panel: ERPs elicited by standard and target stimuli. First panel: ERPs elicited by standard and target stimuli. Second to fourth panels: ERP difference waveforms (target minus standard) showing the P3b evoked by sound changes to azimuth, near, and far targets. Shaded areas beyond the waveforms refer to the range of +/- one standard error. Topographic maps refer to the point in time when the area of the respective component reaches 50 percent (FAL).

Table S11. Pairwise post-hoc analysis of the effect of target dimension (azimuth, near, far) on MMN and P3b mean amplitudes and fractional area latencies (FAL). T- and p-values were corrected based on the jackknifing procedure. P-values were corrected for multiple testing using False Discovery Rate correction.

| | **MMN Mean Amplitude** | | | | **MMN FAL** | | | | **P3b Mean Amplitude** | | | | **P3b FAL** | | | |
|---|---|---|---|---|---|---|---|---|---|---|---|---|---|---|---|---|
| | ***$M_1$ ($SD_1$)*** | ***$M_2$ ($SD_2$)*** | ***T*** | ***p*** | ***$M_1$ ($SD_1$)*** | ***$M_2$ ($SD_2$)*** | ***T*** | ***p*** | ***$M_1$ ($SD_1$)*** | ***$M_2$ ($SD_2$)*** | ***T*** | ***p*** | ***$M_1$ ($SD_1$)*** | ***$M_2$ ($SD_2$)*** | ***T*** | ***p*** |
| **Azimuth vs. Near** | -0.98 (0.21) | -0.25 (0.24) | 2.39 | .064 | 147.82 (5.97) | 172.24 (35.88) | -0.15 | .879 | 3.28 (0.44) | 2.72 (0.42) | 0.69 | .741 | 449.78 (12.72) | 526.41 (23.16) | -1.11 | .275 |
| **Azimuth vs. Far** | -0.98 (0.21) | -0.31 (0.26) | 1.98 | .080 | 147.82 (5.97) | 211.56 (13.67) | -0.64 | .879 | 3.28 (0.44) | 2.59 (0.40) | 1.15 | .741 | 449.78 (12.72) | 623.74 (29.74) | -1.28 | .275 |
| **Near vs. Far** | -0.25 (0.24) | -0.31 (0.26) | 0.12 | .908 | 172.24 (35.88) | 211.56 (13.67) | 0.49 | .879 | 2.72 (0.42) | 2.59 (0.40) | -0.10 | .922 | 526.41 (23.16) | 623.74 (29.74) | 1.38 | .275 |
